# Interaction with hyaluronan matrix and miRNA cargo as contributors for in vitro potential of mesenchymal stem cell-derived extracellular vesicles in a model of human osteoarthritic synoviocytes

**DOI:** 10.1186/s13287-019-1215-z

**Published:** 2019-03-29

**Authors:** Enrico Ragni, Carlotta Perucca Orfei, Paola De Luca, Gaia Lugano, Marco Viganò, Alessandra Colombini, Federico Valli, Daniele Zacchetti, Valentina Bollati, Laura de Girolamo

**Affiliations:** 1 IRCCS Istituto Ortopedico Galeazzi, Laboratorio di Biotecnologie Applicate all’Ortopedia, Milan, Italy; 2 IRCCS Istituto Ortopedico Galeazzi, Chirurgia Articolare Sostitutiva e Chirurgia Ortopedica (CASCO), Milan, Italy; 3 Division of Neuroscience, IRCCS San Raffaele Scientific Institute, Milan, Italy; 4 EPIGET - Epidemiology, Epigenetics and Toxicology Lab, Department of Clinical Sciences and Community Health, Università degli Studi di Milano, Milan, Italy

**Keywords:** Adipose-derived mesenchymal stem cells, Synoviocytes, Extracellular vesicles, Hyaluronan coat, miRNA, Osteoarthritis

## Abstract

**Background:**

Osteoarthritis (OA) is the most prevalent joint disease, and to date, no options for effective tissue repair and restoration are available. With the aim of developing new therapies, the impact of mesenchymal stem cells (MSCs) has been explored, and the efficacy of MSCs started to be deciphered. A strong paracrine capacity relying on both secreted and vesicle-embedded (EVs) protein or nucleic acid-based factors has been proposed as the principal mechanism that contributes to tissue repair. This work investigated the mechanism of internalization of extracellular vesicles (EVs) released by adipose-derived MSCs (ASCs) and the role of shuttled miRNAs in the restoration of homeostasis in an in vitro model of human fibroblast-like synoviocytes (FLSs) from OA patients.

**Methods:**

ASC-EVs were isolated by differential centrifugation and validated by flow cytometry and nanoparticle tracking analysis. ASC-EVs with increased hyaluronan (HA) receptor CD44 levels were obtained culturing ASCs on HA-coated plastic surfaces. OA FLSs with intact or digested HA matrix were co-cultured with fluorescent ASC-EVs, and incorporation scored by flow cytometry and ELISA. ASC-EV complete miRNome was deciphered by high-throughput screening. In inflamed OA FLSs, genes and pathways potentially regulated by ASC-EV miRNA were predicted by bioinformatics. OA FLSs stimulated with IL-1β at physiological levels (25 pg/mL) were treated with ASC-EVs, and expression of inflammation and OA-related genes was measured by qRT-PCR over a 10-day time frame with modulated candidates verified by ELISA.

**Results:**

The data showed that HA is involved in ASC-EV internalization in FLSs. Indeed, both removal of HA matrix presence on FLSs and modulation of CD44 levels on EVs affected their recruitment. Bioinformatics analysis of EV-embedded miRNAs showed their ability to potentially regulate the main pathways strictly associated with synovial inflammation in OA. In this frame, ASC-EVs reduced the expression of pro-inflammatory cytokines and chemokines in a chronic model of FLS inflammation.

**Conclusions:**

Given their ability to affect FLS behavior in a model of chronic inflammation through direct interaction with HA matrix and miRNA release, ASC-EVs confirm their role as a novel therapeutic option for osteoarthritic joints.

**Electronic supplementary material:**

The online version of this article (10.1186/s13287-019-1215-z) contains supplementary material, which is available to authorized users.

## Background

Osteoarthritis (OA) is the most prevalent joint disease, affecting 250 million people worldwide. The number of affected people aged 60 years old and above is predicted to double by 2050 and more than triple by 2100 [1], making this pathology among the present and future main causes of disability [2]. OA is characterized by osteophyte formation, sub-chondral bone sclerosis, cartilage degradation, calcification of ligaments, and synovial inflammation. Crosstalk between the joint tissues such as cartilage and synovial membrane, especially at the cellular levels (chondrocytes and synoviocytes) within an immune inflammatory network, can promote and accelerate synovitis and cartilage degradation. Current treatments are essentially symptomatic to handle pain and swelling, and mainly rely on antalgics and non-steroid anti-inflammatory drugs (NSAIDs) [3]. To date, no options for effective tissue repair and restoration are available, since only total knee arthroplasty, implying a total replacement of the joint, provides a definitive solution.

Mesenchymal stem cells (MSCs) have emerged as an encouraging tool in regenerative approaches due to self-renewal and immunomodulatory properties, together with their capacity for multipotent differentiation into several lineages including osteoblasts, adipocytes, and chondrocytes [4]. MSCs can be isolated from almost all tissues including a perivascular area [5]. Due to a minimally invasive harvesting procedure together with low morbidity, adipose tissue (AD) is currently selected among the most widely used sources. Further, stromal vascular fraction (SVF), derived from AD, is routinely used with high safety and efficacy in patients with OA [6], with resident adipose tissue-derived MSCs (ASCs) considered as major means by which SVF mediate anti-inflammatory, anti-apoptotic, anti-fibrotic, angiogenic, mitogenic, and wound healing properties [7]. Under these premises, MSCs impact on joint diseases has been explored, and the efficacy of autologous or allogenic MSCs has been demonstrated in both animal studies and clinical trials [8–10], opening the possibility of their use as new therapeutic agents, although their mode of action remains to be fully deciphered. In this view, in recent years, it clearly emerged that, apart the paradigm of direct trans-differentiation, MSCs mainly modulate the injured tissue environment to orchestrate regenerative processes by the secretion of anti-apoptotic, anti-inflammatory, proangiogenic, immunomodulatory, and anti-scarring protein- and nucleic acid-based factors [11, 12], both free and conveyed within released extracellular vesicles (EVs) [13].

EVs are a heterogeneous population of nanoparticles released by all cell types and involved in cell-to-cell communication. EVs consist of microvesicles (100–500 nm in size) and exosomes (50–150 nm), both composed of a lipid bilayer, yet exosomes are peculiar by being formed within endosomes [11]. MSC-EVs have been demonstrated to carry proteins, cytokines, and nucleic acids capable to be translated into functional factors in recipient cells [14–16]. In this path, recent works showed that MSC-EVs, similar to MSCs, have both anti-inflammatory and regenerative potential in cell types and tissues usually heavily affected by OA condition. In particular, ASC-EVs were able to correct OA osteoblast metabolism by downregulating senescence-associated β-galactosidase activity and reducing the production of inflammatory mediators [17]. Similarly, in inflamed OA chondrocytes, ASC-EVs reduced the production of inflammatory mediators, decreased the release of metalloproteases activity, and increased the production of the anti-inflammatory cytokine IL-10, therefore resembling the anti-inflammatory and chondroprotective effects of ASCs [18]. Intriguingly, those recent findings resemble data previously published for EVs obtained from MSCs isolated or derived from other tissues or cell types that are more difficult to obtain (bone marrow) or under technical (induced pluripotent stem cells, iPS) or ethical (embryonic stem cells) issues. As an example, bone marrow-derived MSC-EVs could increase the expression of typical chondrocyte markers (type II collagen, aggrecan) while inhibiting catabolic (MMP-13, ADAMTS5) and inflammatory (iNOS) ones in vitro, and protected mice from developing OA in vivo [19]. Consistently, EVs from embryonic stem cell-derived MSCs were shown to balance in vivo the synthesis and degradation of chondrocyte extracellular matrix [20]. Further, exosomes derived from iPS-derived MSCs successfully decreased OA symptoms in vivo [21].

Therefore, these pioneer reports opened the possibility of a future use of ASC vesicles to manage OA [22], taking into account that EVs may present considerable advantages over cells for manufacturing, storage, handling, product shelf life, and potential as a ready-to-go biologic product. In fact, the use of mesenchymal stem cells was reported to have some drawbacks, such as issues related to ectopic tissue formation, infusional toxicity caused by cells lodged in the pulmonary microvasculature, and cellular rejection or unwanted engraftment [23]. However, none of these studies focused attention on the EV–target cell interaction, a crucial factor to be dissected and improved for future strategies aimed at obtaining more effective medicinal products. Further, no direct correlation between EV cargo and observed molecular outcomes has been clearly ruled out. In an attempt to solve these major issues, the first aim of the present study was to characterize the mechanisms underlying ASC-EV uptake in synoviocytes, exploring new strategies to improve the mechanism. The second goal was to dissect miRNA content of EVs to predict and further confirm their capacity to modulate target cells under continuous inflammation, an in vitro experimental setting resembling in vivo conditions.

## Methods

### Donors

The study was carried out at IRCCS Istituto Ortopedico Galeazzi (IOG). The synovial membranes were obtained from the waste material of three female patients (average age 72 ± 7 years) who had undergone total knee arthroplasty. The adipose tissue samples were obtained from waste material of three female donors (average 54 ± 8 years) who underwent liposuction for regenerative medicine purposes (treatment of Achilles tendinopathy) at the RE.GA.IN™ Centre of IOG. All the samples, both synovial membranes and adipose tissues, were collected under Institutional Review Board approval (M-SPER-015 - Ver. 2 - 04.11.2016) and after patient informed consent collection.

### Cell isolation and expansion

Adipose-derived MSCs (ASCs) were obtained as previously described [24]. Briefly, the adipose tissue was enzymatically digested (37 °C, 30 min) by 0.075% *w*/*v* type I collagenase (Worthington Biochemical Co., Lakewood, NJ, USA). After digestion, samples were filtered through a cell strainer (100 μm) and centrifuged (1000×*g*, 5 min). Released cells were seeded at 5 × 10^3^ cells/cm^2^ in DMEM supplemented with 10% FBS (GE Healthcare, Piscataway, NJ, USA) and pen/strepto (Life Technology, Carlsbad, CA, USA). Synovial membranes were minced in small pieces and enzymatically digested (37 °C, 3 h) by 0.25% *w*/*v* type I collagenase (Worthington Biochemical Co., Freehold, NJ, USA). After digestion, samples were filtered through a cell strainer (100 μm) and centrifuged (376×*g*, 5 min). Cells were seeded at 5000 cell/cm^2^ density and fibroblast-like synoviocytes (FLSs) selected for plastic adherence [25]. ASCs and FLSs were cultured in DMEM supplemented with 10% FBS and pen/strepto. All cell types were maintained in an incubator at 37 °C in a humidified atmosphere with 5% CO_2_ and used for the following experiments between passages 3 and 5.

### Immunophenotype characterization

Immunophenotype analysis was performed on three different representative populations for each cell type. For ASCs, the experiments were conducted at passage 3 and cells incubated for 10 min at 4 °C in the dark with anti-human antibodies: CD90-FITC, CD44-PE CD73-PE, CD105-PerCP-Vio700, CD34-PE-Vio770, and CD45-PE-Vio770 (Miltenyi Biotec, Bergisch Gladbach, Germany). For FLSs, analyses were conducted at passages 0 and 1, and cells stained for 10 min at 4 °C in the dark with anti-human antibodies: CD73-PE (Miltenyi) and CD14-FITC (Ancell Corporation, Bayport, MN, USA). Unstained samples for each population were used as negative controls, and data were acquired by the CytoFLEX flow cytometer (Beckman Coulter, CA, USA) collecting a minimum of 50,000 events.

### ASC senescence assessment

Thirty-three micromolar 5-dodecanoylaminofluorescein di-β-D-galactopyranoside (C_12_FDG) (Sigma-Aldrich, St. Louis, MO, USA) was added to the medium, and ASCs were incubated for 1 h at 37 °C, 5% CO_2_. After incubation, ASCs were washed with PBS, suspended in 200-μl PBS, and analyzed immediately using a CytoFLEX flow cytometer [26]. Unstained cells were used as negative controls. C_12_FDG-positive events were measured on the FITC detector collecting a minimum of 50,000 events.

### Cell viability assay

Evaluation of viability was determined as previously described [27]. Briefly, annexin V Alexa Fluor® 488 was added to ASCs and the mixture was left at RT in the dark for 20 min. ASCs were centrifuged and suspended pellet incubated with propidium iodide at RT in the dark for 5 min. The stained cells were then loaded onto the Tali® Image-Based Cytometer (Thermo Fisher Scientific, Waltham, MA, USA) and fluorescence scored.

### Red blood cell exclusion assay

Red blood cells were suspended in PBS containing 0.1% BSA, washed three times and suspended in PBS, added to the culture, allowed to settle on FLSs for 15 min at 37 °C, and examined by IX71 Olympus inverted microscope (Olympus, Tokyo, Japan).

### Microscopy of live cells to visualize FLS protrusions

FLSs at 60% confluence were washed with PBS and incubated with anti-human CD73-PE antibody (Miltenyi, 1:10,000 dilution in PBS) for 10 min at 37 °C in the dark. Then, cells were washed twice with PBS and fluorescent images were obtained with an IX71 Olympus inverted microscope.

### ASC-EV isolation

To isolate extracellular vesicles, DMEM without FBS was used and conditioning performed for 24 h. Collected conditioned medium was subjected to differential centrifugation as described in [28] with few modifications. In short, floating cells were removed by centrifugation at 376×*g* for 15 min. Collected supernatant was subsequently centrifuged at 1000×*g* for 15 min, followed by 2000×*g* for 15 min and two sequential centrifugations at 4000×*g* for 15 min. All steps have been performed at 4 °C. Vesicles were finally pelleted by ultracentrifugation at average 100,000×*g* for 4 h at 4 °C in 70Ti rotor (Beckman) and further washed with PBS with same centrifugal force and temperature for 1 h. Pellet was dissolved in PBS and stored at 4 °C for use within 2 days or at − 80 °C for prolonged storage. To obtain fluorescent EVs, after debris cleaning, CFDA-SE (Sigma-Aldrich) was added directly to conditioned medium at 10-μM final concentration and staining was performed for 1 h at 37 °C in the dark before ultracentrifugation. Thereafter, EV pellet obtained as previously described was suspended in PBS and stored at 4 °C or − 80 °C.

### Nanoparticle tracking analysis

Nanoparticle tracking analysis (NTA) was carried out as previously described using the NanoSight system (NanoSight; Wiltshire, UK, www.malvernpanalytical.com/en/) on EVs suspended in PBS [16]. Vesicles were 50 fold diluted in PBS and visualized by light scattering using a conventional optical microscope aligned perpendicularly to the beam axis. NTA software tracked between frames the Brownian motion of individual vesicles calculating the total concentration and size through the application of Stokes-Einstein equation.

### Transmission electron microscopy

Five microliters of purified EVs, corresponding to approximately 500 × 10^6^ particles, were absorbed on Formvar carbon-coated grids for 10 min. The drops were then blotted with filter paper and negatively stained with 2% uranyl acetate (5 μl) in aqueous suspension for 10 min. Excess of uranyl was removed by touching the grid to a filter paper. The grid was dried at room temperature. Grids were examined with a transmission electron microscope (TALOS L120C Thermo Fisher Scientific, Waltham, MA, USA) at 120 kV.

### Dedicated flow cytometry instrument settings

Flow cytometry data on EVs were obtained using a CytoFLEX flow cytometer (Beckman Coulter). Flow cytometer calibration was first verified using a reference bead mix (Biocytex, Marseille, France) composed of a FITC fluorescent mixture of spheres with diameters of 100 nm, 300 nm, 500 nm, and 900 nm. Gains were set at FSC = 106, SSC = 61, FITC = 272, PE = 116, and PC7 = 371. FITC threshold was set at 500 to include 100 nm beads and some smaller debris in the FITC channel.

### Labeling of EVs with antibodies and immunophenotype

Purified carboxyfluorescein succinimidyl ester (CFSE)-positive EVs were incubated for 20 min at 4 °C in the dark with mouse anti-human CD44-PE and CD63-PE-Vio770 (Miltenyi) [16]. Before use, each antibody was centrifuged for 30 min at 4 °C at 16,000×*g* to remove aggregates and debris. Prior to data acquisition, the samples were 1:10 diluted with 0.22-μm triple-filtered PBS. Unlabeled EVs and PBS-diluted Abs were used as negative controls. Data were acquired with calibrated CytoFlex for 120 s at a low flow rate.

### Determination of the number of ASC-EVs incorporated in FLSs by ELISA

CFSE-labeled EVs were maintained in the dark at 4 °C to avoid photobleaching and administrated to FLSs with a 10,000:1 to 160,000:1 EVs/cell ratio. Cells were cultured in the dark. After 24 h, cells were washed, detached, and suspended in PBS to a concentration of 1000,000 cell/ml. All steps were performed in the dark. 0.1 ml was analyzed by ELISA (VICTOR™ X3 Multilabel Plate Reader, Perkin Elmer, Turku, Finland) in a 96-well plate with a 495-nm filter for FITC excitation and a 520-nm filter for emission. Cells treated with unlabeled EVs were used as negative control to subtract fluorescence background. In separate wells, incremental amounts of CFSE-labeled ASC-EVs, determined by NTA, were suspended in 0.1 ml PBS and scored for fluorescence. Signal intensity of EV-treated cells after background subtraction was compared to CFSE-labeled EV fluorescence to determine the number of incorporated EVs. The same experimental approach was followed also for kinetics of incorporation over time with ELISA reads at 1, 3, 6, 12, and 24 h. Similarly, this protocol was used both in the presence of 2 mg/mL soluble HA (Acros Organics, Pittsburgh, PA, USA) and after FLS-associated HA coat removal. For this last condition, before EV supplementation, FLS were treated for 12 h with 1-mM 4-methylumbelliferone (4-MU) (Sigma-Aldrich), an HA synthesis inhibitor, followed by 1-h digestion with 10 U/mL hyaluronidase (Sigma-Aldrich). Finally, EV incubation was performed in the presence of 4-MU to avoid HA matrix neosynthesis. In both cases (soluble HA or HA coat removal), instead of comparison with labeled EVs to calculate the number of uptaken EVs, ASC-EV-treated FLS were used as a control to determine increased or decreased incorporation rates.

### Assessment of CFSE-labeled EV transfer to FLSs by flow cytometry or microscopy

Labeled vesicles were incubated with FLSs at a 100,000:1 (EVs:cell) ratio and maintained in the dark. PBS was used as a control. Cells were observed after 24 h by flow cytometry with a CytoFlex instrument (Beckman Coulter) or, after Hoechst staining of nuclei and CD73 labeling with a-CD73-PE Ab (Miltenyi), 10 min at 37 °C in the dark, under a Leica TCS SP5 Laser Scanning Confocal microscope (Leica Microsystems, Wetzlar, Germany).

### EV release by FLSs

EV release by FLSs (FLS-EVs) and nanoparticles NTA quantification were performed as described for ASCs. To detect CFSE-positive FLS-EVs after fluorescent ASC-EV incubation, FLSs were washed three times with PBS and medium without FBS added for 24 h. Then, supernatant was directly analyzed by calibrated CytoFlex in the FITC channel. Supernatants of FLSs that were not treated with ASC-EVs were used as a negative control. To check the presence of ASC-EVs entrapped in FLS HA matrix, FLSs were washed three times with PBS and digested for 10 m at 37 °C with 10 U/mL hyaluronidase to reduce at minimum the active secretion of fluorescent FLS-EVs. The supernatant of digestion was analyzed by calibrated CytoFlex using hyaluronidase digestion of FLSs that were not co-cultured with ASC-EVs as a negative control.

### Screening of EV-embedded miRNA expression

EVs from three independent population of ASCs were collected and pellets stored at − 80 °C. miRNAs were isolated from frozen EV pellets by using the miRNeasy Kit and RNeasy CleanUp Kit (Qiagen, Hilden, Germany). miRNAs were prepared by standard reverse transcription (RT) and preamplification procedures, followed by real-time RT-PCR analysis with the QuantStudio™ 12 K Flex OpenArray® Platform (QS12KFlex) as previously described [29]. Gene Expression Suite Software (Life Technologies) was used to process miRNA expression data from the miRNA panel. After data cleaning, 267 miRNAs with C_RT_ value < 27 and AmpScore > 1.1 were selected since amplified in all ASC-EVs under analysis. The global mean was selected as normalization method due to the high correlation between samples [30]. miRNA expression was determined using the relative quantification 2^−ΔCRT^.

### Pathway analysis

Shared ASC-EV miRNAs were cleaned from those already expressed in inflamed FLSs (https://www.ncbi.nlm.nih.gov/gds, GSE91026) [31], and unique candidates analyzed by miRWalk 3.0 database to identify mRNA targets (http://mirwalk.umm.uni-heidelberg.de/) [32], focusing only on miRNA–gene interactions that were validated experimentally (https://bio.tools/mirtarbase) [33]. The list of target genes, cleaned from those that were not found to be expressed in inflamed FLSs (GSE49604) [34], was used as input for the DAVID Functional Annotation Clustering tool (https://david.ncifcrf.gov/home.jsp) [35, 36]. The tool provides analysis of annotation content and gene ontology term enrichments, to highlight the most relevant GO terms associated with a gene list. The enrichment score is a geometric mean of the member’s *p* values in a -log scale within an annotation cluster.

### FLS inflammation and gene expression analysis

FLSs were treated for 16 days with 25 pg/mL IL-1β (Peprotech, Rocky Hill, NJ, USA), with medium change every 2 days to avoid cytokine depletion. At time 6 days (time point 0 since EV treatment), ASC-EVs (100,000:1 ratio) were added to inflamed FLSs for the remaining 10 days (time point 10 days). EVs were freshly supplemented every 2 days. At time points 0, 2, and 10 days, total RNA was isolated by Trizol (Sigma-Aldrich) followed by RNeasy mini kit combined with the RNase-free DNase on-column treatment (Qiagen GmbH, Hilden, Germany) [37]. First-strand cDNAs were synthesized using the iScript cDNA synthesis kit (Bio-Rad Laboratories, CA, USA). Primers for *HAS1-2-3*, *MMP1-3-13*, *CCL25*, *IL-6*, *CXCL8*, and *ICAM1* were designed using the NCBI Primer Designing Tool (http://www.ncbi.nlm.nih.gov/tools/primer-blast/). *TBP* was used as a reference. Primer sequences will be provided upon request. Quantifications were performed using “PowerUp SYBR Green Master Mix” (Applied Biosystems, Warrington, UK) and Comparative Ct Method in a StepOne Plus PCR Real Time Instrument (Applied Biosystems). For time 0 analysis, non-inflamed FLSs were used as control whereas for time points 2 and 10, IL-1β-treated FLSs were selected. ^–ΔΔCt^ was used to score differential expression.

### ELISA assays

Concentrations of soluble CCL2, CCL5, and IL-6 in cell culture media collected at time point 10 days after EV supplementation were determined by commercially available ELISA according to the manufacturers’ instructions (PeproTech, Rocky Hill, NJ, USA). Dilutions were made to have absorbance readings within the standard curve values. The detection ranges were 31–2000 pg/mL for CCL5, 15–1000 pg/mL for CCL2, and 31–2000 pg/mL for IL-6.

### Statistical analysis

Statistical analysis was performed using GraphPad Prism Software version 5 (GraphPad, San Diego, CA, USA). Normal data distribution was assessed by the Kolmogorov–Smirnov normality test. Student’s *t* test and Wilcoxon signed-rank test were used to compare data. The level of significance was set at *p* value < 0.05.

## Results

### ASC isolation and EV characterization

**Fig. 1:**
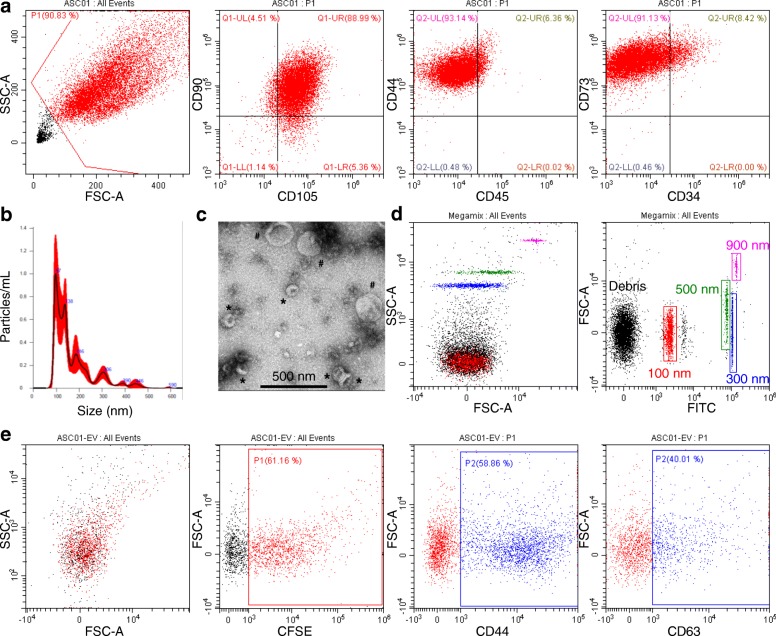
Characterization of ASCs and ASC-EVs. **a** Representative dot plots of MSC (CD44-73-90-105) and hemato-endothelial (CD34-45) markers in ASCs. One representative cell isolate is shown. **b** NTA analysis of representative cell culture supernatant showing the presence of particles in the range between 50 and 500 nm. **c** Electron micrographs of ASC-derived small size (asterisk: possible exosomes) and large size (number sign: microvesicles) vesicles. **d** Setting up the EV-dedicated flow cytometer. The resolution of the reference bead mix indicates the flow cytometer performance in light scattering at default settings. Left cytogram shows side scatter (SSC) versus forward scatter (FSC). Right cytogram depicts FSC versus 535/35 (green fluorescence triggering) channel. Four fluorescent populations (100, 300, 500, and 900 nm) were resolved from the instrument noise. **e** CFSE-labeled ASC-EVs stained with stem cell (CD44) and EV (CD63) markers. ASC-EVs were first gated in the FITC channel (CFSE positive)

Isolated adipose-derived MSCs (ASCs) expressed mesenchymal surface antigens CD73, CD90, CD105, and CD44 and were negative for hemato-endothelial markers such as CD45 and CD34, confirming their identity [38] (Fig. 1a). ASCs were cultured up to five passages at most and consistently showed a homogeneous fibroblastic morphology and no signs of senescence (data not shown). For EV isolation, ASCs were cultured for 24 h in DMEM without FBS (to avoid vesicles present in the serum), with no influence on cell viability (DMEM+FBS 1.7 ± 1.2% PI+ vs DMEM 2.7 ± 1.5% PI+, *n* = 3, *p* = ns) although cell size significantly reduced (DMEM 8.7 ± 0.6 μm vs DMEM+FBS 12.7 ± 0.6 μm, *n* = 3, *p* < 0.005, size of cells after trypsinization). After EV collection, nanoparticle tracking analysis (NTA) has been used to check both the number and size of secreted particles. ASCs were able to release 10,500 ± 1500 vesicles per cell per day. EVs ranged in size from 40 to 50 nm to 300–400 nm (Fig. 1b), as confirmed by transmission electron microscopy (Fig. 1c). The mean and mode sizes were 158 ± 12 nm and 101 ± 8 nm, respectively. Eighty percent of vesicles ranged between 92 ± 6 nm and 246 ± 19 nm. Interestingly, 50% of events were collected up to the size of 144 ± 17 nm, suggesting a high recovery of particles consistent with size features of exosome or, as recently defined by a position statement of the International Society for Extracellular Vesicles, “small EVs” [39]. To confirm these data, EVs were also analyzed by flow cytometry, after instrument calibration with 100, 300, 500, and 900 nm fluorescent beads (Fig. 1d). CFDA-SE staining was performed on EVs and fluorescent events detected (Fig. 1e). By comparison with calibration beads, 34 ± 10% of CFDA-SE-labeled EVs fell in the range ≤ 100 nm with respect to 35 ± 11% scored by NTA, and 59 ± 11% ranged 100–300 nm compared to 62 ± 11%. Finally, flow cytometry analysis confirmed the presence of both MSC (CD44) and EV (CD63) markers (Fig. 1e), further validated by Western Blot analysis (data not shown). CD44 positivity confirmed recent data about its role in defining MSC-EV surface signature [40].

### Synoviocyte isolation and ASC-EV incorporation

**Fig. 2:**
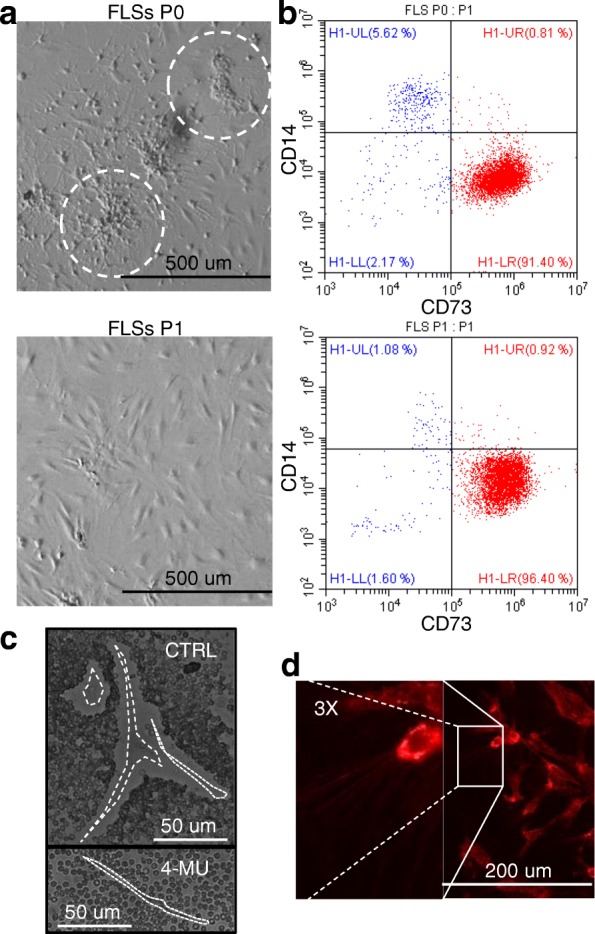
Characterization FLSs. **a**, **b** Representative FLS population at passage 0 (P0) and 1 (P1) shows the initial presence and steady reduction of CD14+ monocyte with enrichment of CD73+ events. **c** Synovial cells (CTRL) with added red blood cells to demonstrate clear pericellular zones. As control of HA coat involvement in erythrocyte exclusion, synovial cells pre-treated for 24 h with 4-MU to block HA coat synthesis are shown (4-MU). Bright field. **d** FLSs stained for CD73 surface marker shows thin lines representing cell protrusions

After isolation of the synovial membrane and collagenase digestion, at passage 0, two cell types were selected, one spindle-shaped (FLSs) and one with polygonal-star morphology (macrophages) (Fig. 2a). Flow cytometry analysis confirmed the presence of a mixed population composed of either CD73 (FLS marker) or CD14 (macrophage) positive cells (Fig. 2b). Steadily at passage 1, polygonal-star CD14+ macrophages almost disappeared (Fig. 2a, b). To further confirm FLS identity, the presence of a thick pericellular coat composed of hyaluronan (HA) was detected by exclusion of sedimenting erythrocytes, as first shown in 1968, and corroborated by erythrocytes proximity after HA coat synthesis inhibition by 4-MU (Fig. 2c) [41]. HA coat resulted to be 7.5 ± 2.5-μm thick. The HA scaffold has been suggested to be assembled by ongoing synthesis at the level of plasma membrane protrusions in fibroblasts and chondrocytes, known for their endogenously active HA deposition [42]. Consistently, fluorescence microscopy on CD73 stained FLSs showed the presence of membrane protrusions along the cell surface (Fig. 2d).

**Fig. 3:**
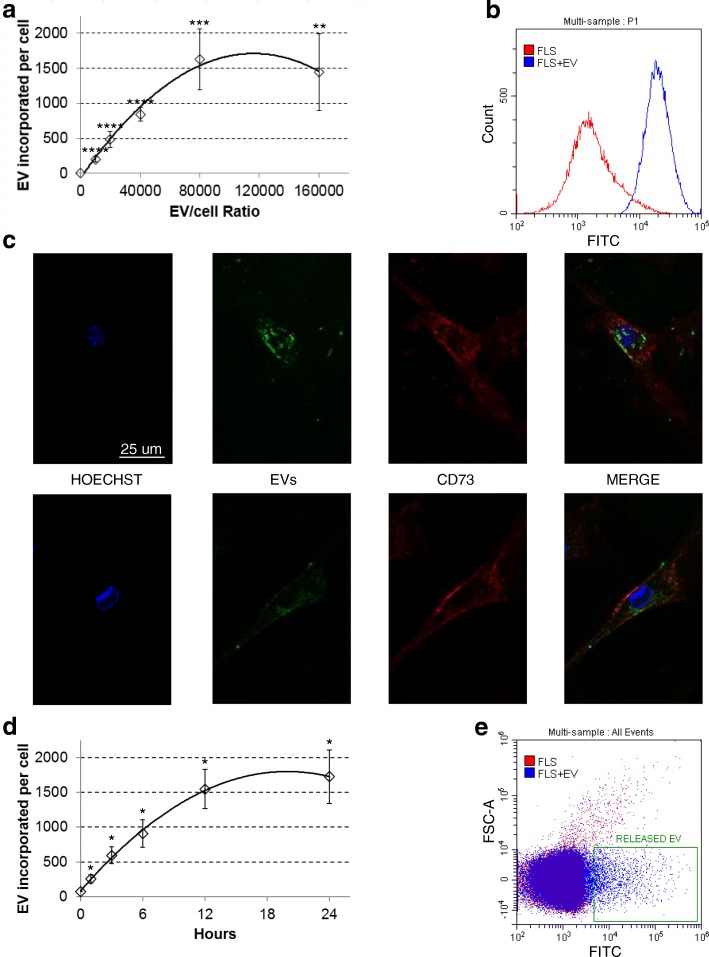
ASC-EVs are taken up by synoviocytes. **a** CFSE-EVs incorporated by FLSs after 24-h co-culture at different EV:FLS ratios. Number of incorporated EVs has been calculated comparing FLS fluorescence with signal given by a pre-determined number of isolated CFSE-labeled EVs (Quantification of data from three independent FLSs, each incubated with three independent ASC-EVs, is shown as mean ± SD. ****p < 0.0001; ***p < 0.001; **p < 0.01). **b** Flow cytometry of CFSE-EV-treated FLSs at 100,000:1 ratio showing that all cells incorporate fluorescent vesicles. **c** Confocal microscopy images of FLSs after 24-h co-culture with labeled ASC-EVs. Uptaken EVs localize in perinuclear areas. **d** Kinetics of EV incorporation (100,000 EVs per FLS, *n* = 3 independent experiments, each FLS incubated with a (1:1:1) mix of ASC-EVs from three independent ASCs. Data are presented as mean ± SD. *p ≤ 0.05). **e** FLSs, after 24-h incubation with CFSE-EVs, are able to release fluorescent vesicles (box). Representative plot of three independent analyses

Thus, ASC-EV uptake mechanisms were investigated. Increasing concentrations of CFSE-labeled ASC-EVs were added to cultured FLSs and incubated for 24 h. EVs to cell ratio ranged from 10,000:1 to 160,000:1, meaning, due to the approximate value of 10,000 EVs released by single ASC, an equivalent ASC:FLS ratio from 1:1 to 16:1. EV uptake was dose-dependent, with saturation around 100,000 EVs per FLS (Fig. 3a). Flow cytometry analysis showed that, after 24 h, all cells were CFSE positive (Fig. 3b) with EVs mainly localized in the perinuclear area (Fig. 3c). A similar result was also obtained after ex vivo incubation of a synovial membrane with labeled EVs and further synoviocyte isolation, confirming the ability of ASC vesicles to be incorporated by FLSs in their environment (data not shown). Next, uptake kinetics was monitored. Flow cytometry demonstrated fluorescence gain over time (Fig. 3d) with an increase in fluorescence as fast as 60 min. Notably, linear increase of fluorescence was scored the first 4–6 h, with saturation at around 24 h (Fig. 3d). A similar progression was shown for other and unrelated cell types like bladder cancer [43] or lymphoma [44] cells, implying a conserved kinetics or mechanism.

Since CFSE remains stable within a viable cell for several weeks and passive diffusion out the cell is very slow, after EV cargo release either into the cytoplasm or the endosomal compartment, the possibility of CFSE clearance via secreted vesicles was explored. FLSs were able to release 23,500 ± 500 EVs/cell with mean and mode sizes of 153 ± 3 nm and 113 ± 3 nm, respectively. Further, after incubation for 24 h with fluorescent ASC-EVs, in the next 24 h, FLSs released CFSE-positive vesicles (Fig. 3e). At present, it could not be estimated whether a small amount of ASC-EVs that may have detached from the cell surface before being incorporated was also detected. From this data, the observed reduction in fluorescence increase over time was likely due to a balance between CFSE uptake and release via EVs, a further proof of effective cargo release. Taken this into consideration, and dealing with a proposed time of approximately 3 h for newly generated EV release [45], ASC-EV uptake in the first 4 h was estimated to be around 4000 incorporated vesicles per day. Altogether these data suggest a continuous EV uptake, a crucial factor in view of using ASC-EVs as medicinal products.

### Hyaluronan coat involvement in ASC-EV uptake

**Fig. 4:**
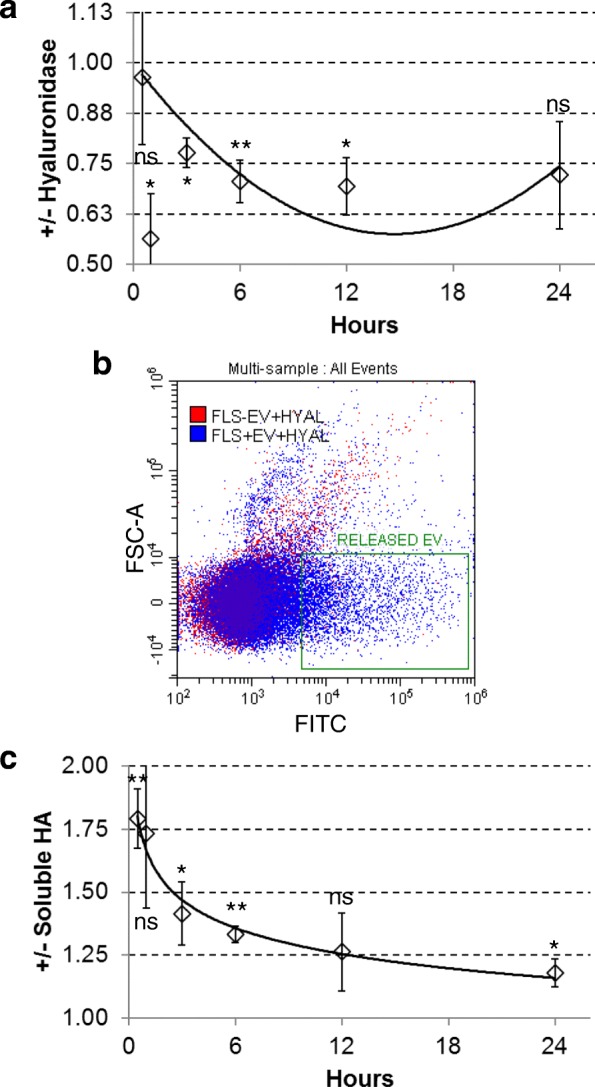
HA coat has a crucial role in EV incorporation. **a** Ratio of ASC-EV incorporation with (+) or without (−) HA coat, after 24 h [100,000 EVs per FLS, *n* = 3 independent experiments, each FLS incubated with a (1:1:1) mix of ASC-EVs from three independent ASCs]. Data are presented as mean ± SD. **p < 0.01, *p ≤ 0.05, ns > 0.05. **b** ASC-EVs release from FLSs after coat digestion with hyaluronidase (+HYAL) for 10 m at 37 °C. Digestion of FLSs with (+EV) or without (−EV) 24-h pre-incubation with ASC-EVs is presented. Released vesicles in the green box. **c** Ratio of ASC-EV incorporation with (+) or without (−) 2 mg/ml soluble high molecular weight HA in growth medium, after 24 h [100,000 EVs per FLS, *n* = 3 independent experiments, each FLS incubated with a mix (1:1:1) of ASC-EVs from three independent ASCs]. Data are presented as mean ± SD. **p < 0.01, *p ≤ 0.05, and ns > 0.05

In this and in a previous work [40], CD44, one of the hyaluronan receptors, has been demonstrated to be highly expressed on both ASC-EVs and bone marrow MSC-EVs, opening the intriguing question whether hyaluronan and its receptors may contribute to vesicle docking and uptake in cells with active HA deposition. First, the role of the coat was investigated. Hyaluronan is a multifunctional glycosaminoglycan up to 10^7^ Da molecular mass produced by three hyaluronan synthases (*HAS1-2-3*). It is a dynamic structure, and after its removal, can be restored by cells in less than 2 h [46]. Thus, to efficiently and permanently remove the HA coat, a combination of 4-methylumbelliferone (4-MU) and hyaluronidase was used. 4-MU is known to both downregulate the expression of *HAS* genes and deplete the HA precursor UDP-GlcUA [47], blocking coat neosynthesis. In this condition, FLSs showed a significant reduction of EV incorporation, up to 30% after 12 h (*p* ≤ 0.05), with impairment already detectable at earlier time points (*p* ≤ 0.05) (Fig. 4a). Diminished uptake persisted at 24 h (30%, *p* = 0.06). These results suggest that cellular hyaluronan coat may act as a sponge increasing the local concentration of EVs around the plasma membrane, thus facilitating their uptake. To corroborate the hypothesis of a “soaked sponge,” FLSs have been treated with hyaluronidase for a short time to reduce at minimum active secretion of CFSE engulfed FLS-EVs. Notably, fluorescent vesicles were recovered (Fig. 4b). Further, since synoviocytes and synovial membrane are in direct contact with synovial fluid that is enriched in soluble high molecular weight hyaluronic acid, this was added to the culture medium during incubation to resemble the environment in the joint. Hyaluronic acid concentration in OA synovial fluid falls within the 1 to 3 mg/ml range [48]; thus, an average value of 2 mg/mL was selected. Interestingly, the presence of soluble HA resulted in a significant increase in vesicle uptake at earlier time points, 1.8 and 1.4 fold at 0.5 and 3 h, respectively (Fig. 4c), with similar behavior to the control only after 24 h (1.2 fold, *p* ≤ 0.05).

### CD44 role in ASC-EV uptake

**Fig. 5:**
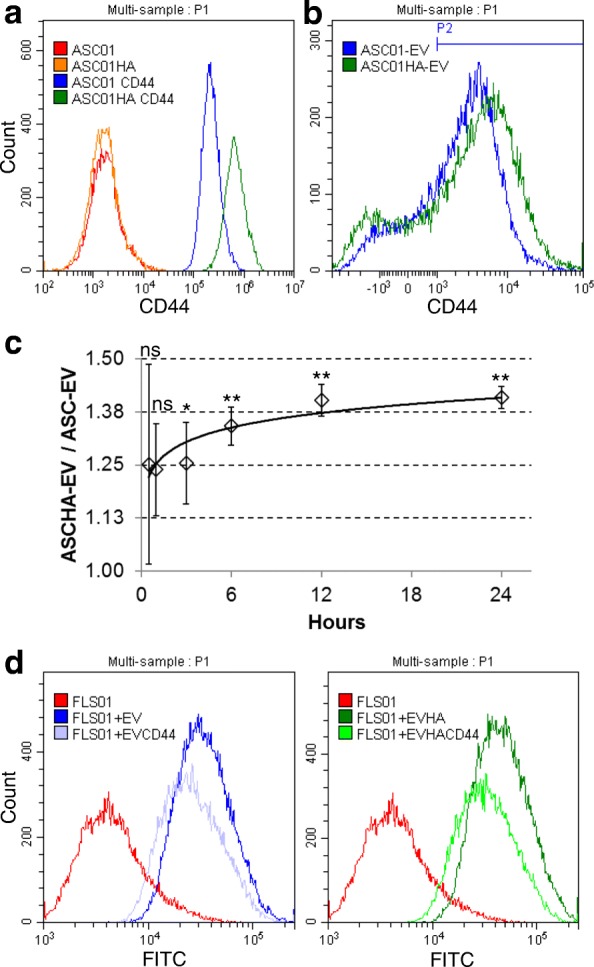
CD44 is involved in ASC-EVs uptake in FLSs. **a** Representative cytogram of CD44 expression in ASCs grown with (ASC01HA) or without (ASC01) HA coating of culture flask. **b** Representative CD44 cytogram of ASC-EVs obtained from “non-primed” ASCs (ASC-EV) or “HA-primed” ASCs (ASCHA-EV) for 24 h. P2 represents CD44-positive particles, after gating on CFSE-positive events as explained in Fig. 1e. **c** Ratio of EV incorporation in FLS between “CD44-boosted” EVs (ASCHA-EV) and “normal” EVs (ASC-EV), after 24 h (100,000 EVs per FLS, *n* = 3 independent experiments, each FLS incubated with a mix of ASC-EVs or ASCHA-EVs from three independent ASCs). Data are presented as mean ± SD. **p < 0.01, *p ≤ 0.05, ns > 0.05). **d** Representative cytogram of FLSs incubated with ASC-EVs (FLS + EV) or with ASC-EVs pre-treated with aCD44 Ab (FLS + EVCD44), and FLSs co-cultured with ASCHA-EVs (FLS + EVHA) or ASCHA-EVs blocked with aCD44 Ab (FLS + EVHACD44)

A possible mean by which ASC-EVs may interact with HA is the presence on their surface of hyaluronan receptors, like CD44. In a recent publication, it was shown that HA coating of culture flask was sufficient to transiently modulate CD44 in mouse bone marrow MSCs [49]. Thus, human ASCs were cultured on HA-coated surfaces for 24 h and CD44 amount scored by flow cytometry (Fig. 5a) and qRT-PCR, resulting in a significant increase of 3.4 (calculated on background subtracted mean fluorescence intensity) and 13.8 fold, respectively, (*n* = 3, *p* ≤ 0.05 for both). Regarding EVs, although the percentage of CD44+ events did not change, the hyaluronan receptor was present at significantly higher levels, with a 30% increase (*n* = 3, *p* ≤ 0.05) (Fig. 5b). On the contrary, CD63+ particles differed neither in their percentage nor in the amount of protein (data not shown), indicating that the observed result is specific for CD44. Moreover, the number of secreted vesicles increased by a 1.5 ± 0.2 factor (*n* = 3, *p* ≤ 0.05). Then, FLSs have been cultured with CD44-boosted fluorescent EVs (Fig. 5c). Faster and persistent increase in fluorescence was scored over time with, notably, CD44-blocking antibody being able to reduce uptake for both normal (− 32 ± 6%, *p* ≤ 0.05) and CD44-boosted (− 36 ± 8%, *p* ≤ 0.05) vesicles (Fig. 5d). These data confirm the importance of hyaluronan matrix and its receptors for the efficient recruitment of EVs, a factor to be taken into considerations for future cell and tissue targets where HA is a predominant component.

### ASC-EV miRNA content

**Fig. 6:**
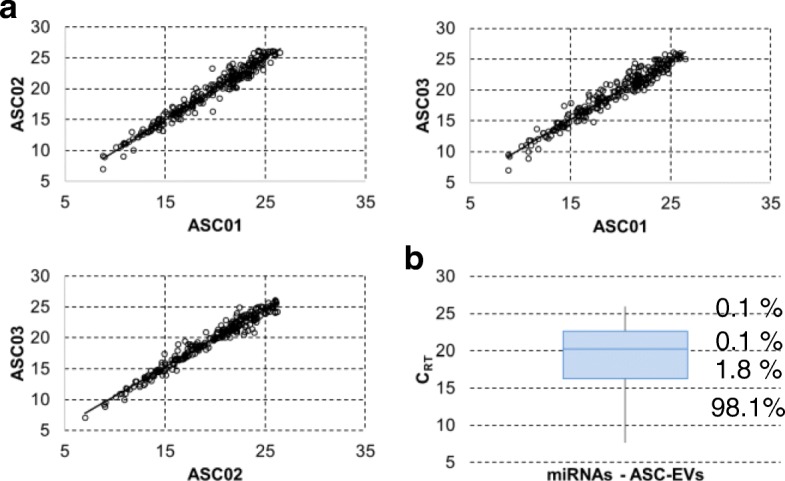
Comparison of genome-wide miRNA expression profiles between ASC-EVs under study. **a** Correlation of miRNA expression levels (normalized *C*_RT_) between the three ASC-EVs under study. **b** Box plot of normalized *C*_RT_ values for shared and averaged 267 miRNAs embedded in ASC-EVs. The percentage of total expression for each quartile is shown. First quartile miRNAs have been used for target mining

Once gone across the HA matrix and entered target cells, EVs must transfer their cargo (nucleic acids, proteins, and lipids) to modify cellular phenotypes and/or exert therapeutic efficacy. In this frame, miRNAs make up an important fraction of EV content, being key contributors to their overall biological function [15]. To avoid focusing on single miRNAs, whole miRNA profiling and bioinformatics has been used to determine the miRNA landscape of EVs from three independent ASCs. A total of 319, 292, and 300 miRNAs were identified in the three populations (ASC01-02-03) [Additional file 1: Table S1]. Collectively, 267 miRNAs were always present in all three EV preparations [Additional file 1: Table S2]. Two hundred seventy-eight miRNAs were in common between ASC01 and 02, 281 between ASC01 and 03, and 276 comparing ASC02 and 03. Moreover, normalized amplification cycles were highly correlated (*R*^2^ ≥ 0.95) (Fig. 6a), suggesting consistent conservation of the cargo composition. Therefore, further analyses were performed with averaged values (with 237 candidates having a SD ≤ 1 C_RT_, and 30 ≤ 2).

To associate a biological function with identified miRNAs, it has to be taken into consideration the recent finding that, even for the most abundant type of miRNA, there is around 1.3 miRNA per MSC vesicle [50]. Thus, on average, 100 EVs would be needed to transfer one copy of a given abundant miRNA [51]. In this view, the top 63 miRNAs falling in the first quartile of expression (Fig. 6b and Additional file 1: Table S3), accounting for 98% of all the EV miRNA content, were analyzed. Out of these 63 molecules, 17 were shown to play important roles in regulating cartilage, synovium, and bone homeostasis, being involved at different levels in the pathology of OA (Additional file 1: Table S4) [52]. Notably, many of these OA-related miRNAs have promoting or inhibiting functions, rendering overall prediction of efficacy uncertain. Therefore, to identify candidates having an unambiguous effect on target cells, basing our search on a public dataset (https://www.ncbi.nlm.nih.gov/gds, GSE91026), miRNAs shown to be already expressed in FLSs under inflammation were excluded [31]. Eleven miRNAs remained as unshared and ASC-EVs unique: hsa-miR-191, hsa-miR-30a-5p, hsa-miR-127, hsa-miR-328, hsa-miR-320, hsa-miR-106a, hsa-miR-331, hsa-miR-138, hsa-miR-296, hsa-miR-28, and hsa-miR-663B. Target mining using miRWalk 3.0 (http://mirwalk.umm.uni-heidelberg.de/), and focusing only on miRNA–gene interactions that were validated experimentally (https://bio.tools/mirtarbase), showed that the 11 selected miRNAs, collectively, may target 503 genes. To increase the power of the in silico prediction, identified genes were scored against the first quartile of expressed transcripts in IL-1β-treated FLSs (GSE49604). Collectively, 84 genes resulted to be potential and consistently expressed FLS targets [Additional file 1: Table S5]. This gene set was further analyzed by the Database for Annotation, Visualization and Integrated Discovery (DAVID), which clusters distinct genes by the pathways in which they are involved (https://david.ncifcrf.gov/home.jsp). The two most significant biological process terms were associated with (a) positive regulation of NF-kappaB transcription factor activity (*p* = 3.7E−3), and the genes enriched in this term included interleukin-6 (*IL-6*), nuclear factor kappa B subunit 1 (*NFKB1*), nucleophosmin (*NPM1*), peroxiredoxin 3 (*PRDX3*), and ubiquitin A-52 residue ribosomal protein fusion product 1 (*UBA52*); (b) cell cycle (*p* = 3.8E−3), including G protein subunit alpha i2 (*GNAI2*), cyclin D1 (*CCND1*), cyclin D2 (*CCND2*), cyclin D3 (*CCND3*), thioredoxin-interacting protein (*TXNIP*), and zinc finger MYND-type containing 11 (*ZMYND11*). Consistently, the most enriched cellular component term was cyclin-dependent protein kinase holoenzyme complex (*p* = 3.8E−5) encompassing *CCND1*, *CCND2*, *CCND3*, and cyclin-dependent kinase 6 (*CDK6*). Moreover, cell cycle resulted to be the most significant term both as a keyword (10 genes, *p* = 1.2E−3) and as KEGG_pathway (five genes, *p* = 7.73E−3). In this frame, the cell division biological process was scored with *p* = 2E−2 and 6 genes [*GNAI2*, *CCND1*, *CCND2*, *CCND3*, *CDK6*, and regulator of chromosome condensation 2 (*RCC2*)]. Finally, crucial components of other pathways activated in OA FLSs resulted to be potential target of ASC-EV miRNAs: *CREB1*, transcription factor of the PI3K–Akt signaling pathway inducing the expression of *VEGF* and the production of reactive oxygen species [53]; notably, the KEGG term PI3K-Akt signaling pathway was scored by DAVID with *p* = 2.12E−2 and 7 genes included (*CREB1*, *CCND1*, *CCND2*, *CCND3*, *CDK6*, *IL-6*, and *NFKB1*). And eventually *SOD2*, mitochondrial superoxide dismutase contributing to scavenging of radicals, that, after an initial burst of expression, under chronic cytokine exposure, as in OA, was reported to downregulate its levels, probably due to escape massive cell death effects [54]. Therefore, altogether, these results predict a beneficial impact of ASC-EVs and their unique miRNA content on pathways activated in OA synovia and synoviocytes.

### ASC-EV effect on FLS inflammatory status

**Fig. 7:**
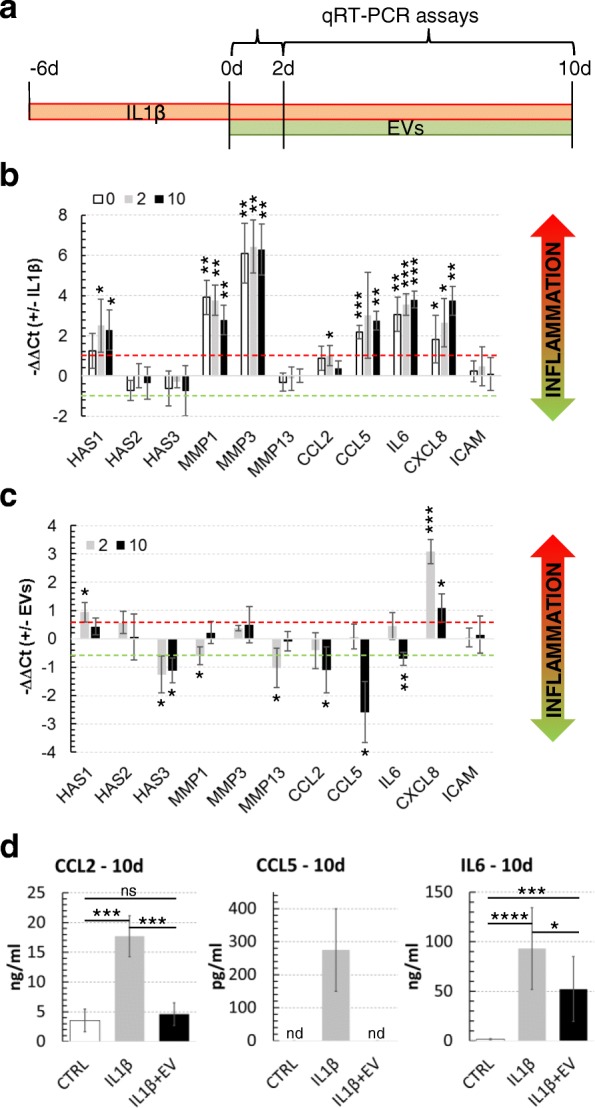
IL-1β and EV effect on FLSs. **a** Experimental plan for IL-1β inflammation and EVs supplementation in FLSs. IL-1β or IL-β + EVs (100,000 EVs:FLS) were freshly added with medium change each 48 h. **b** IL-1β at low concentration activates inflammation markers. Synoviocytes were treated with 25 pg/mL IL-1β and, after 6 days, at time points 0, 2, and 10 days, 11 genes related to inflammation were scored by qRT-PCR. The data are presented as ^−ΔΔCt^ relative to the untreated control for each time point. *p ≤ 0.05, **p < 0.01, ***p < 0.001. **c** EVs are able to reduce secretion of chemokines and cytokines under inflammation stimuli. FLSs treated with 25 pg/mL of IL-1β for 6 days were supplemented with EVs pooled from three ASC supernatants and OA-related genes scored after 2 and 10 days. Quantification of data is shown as mean ± SD and normalized for *TBP*. The data are presented as ^−ΔΔCt^ relative to IL-1β only treated FLSs. *p ≤ 0.05, **p < 0.01, ***p < 0.001. **d** ELISA assays confirm reduction of inflammation-related CCL2/CCL5 chemokines and IL-6 after 10-day EV exposure. Quantification of data is shown as mean ± SD. *p ≤ 0.05, ***p < 0.001, ****p < 0.0001

We lastly assessed the in silico-predicted biological effects of ASC-EVs in an in vitro model of inflamed FLSs. Published protocols to activate FLSs describe the use of interleukin-1 beta (IL-1β) in the range of 1 to 10 ng/ml for 24 h [55, 56]. In this condition, inflammation markers resulted to be strongly upregulated, e.g., *MMP3* up to 300 fold or *IL-6* up to 120 fold [56]. Nevertheless, physiological IL-1β concentration in the synovial fluid is reported to be greatly lower and around 25 pg/mL [57, 58]. Thus, instead of a quick and heavy FLS priming, we opted for a protocol based on a prolonged 16-day IL-1β treatment at physiological levels, with ASC-EVs (EV:FLS ratio of 100,000:1) added 6 days after beginning of IL-1β administration (time 0 for following gene expression analysis) and maintained for additional 10 days (time 10), with medium, IL-1β, and EVs change every 2 days to avoid cytokine degradation or vesicle depletion (Fig. 7a).

First, 11 markers directly associated with FLS activation [59] have been assayed by qRT-PCR in IL-1β-treated samples at time points 0, 2, and 10 days to confirm induction and maintenance of inflammatory phenotype. These markers were *HAS1-2-3*; *MMP1-3-13*, matrix metallopeptidases involved in extracellular matrix degradation; *CCL2-5*, chemokines recruiting innate immune cells such as lymphocytes and monocytes; *IL-6* and *CXCL8* (also named *IL-8*), pro-inflammatory cytokine and chemokine, respectively; and *ICAM1*, an adhesion molecule playing a critical role in maintaining cell homeostasis. Increased transcript levels (− ΔΔCt ≥ 1 with *p* ≤ 0.05 in inflamed samples vs control cells) of *HAS1*, *MMP1-3*, *CCL2-5*, *IL-6*, and *CXCL8*, whose protein products usually distinguish OA joints, were scored (Fig. 7b) [60–62]. Further, *HAS1* increment given by IL-1β is consistent with both published data [63] and observed low molecular weight HA in OA synovial fluid in vivo, since hyaluronan synthase 1 polymerizes small HA chains (2 × 10^5^ to 2 × 10^6^ Da) with respect to *HAS2* that synthesizes very large HA molecules (> 2 × 10^6^ Da) [64]. In this frame, *HAS1*:*HAS2* mRNA ratio increased of 5.9 ± 2.3 fold (*p* < 0.0001). Moreover, *HAS1* increment also agrees with both high levels of HA secretion observed for cultures exposed to IL-1β [65] and the elevated HA amount in OA joints.

Then, the effect of EV supplementation on IL-1β stimulated FLSs at early (2 days) and late (10 days) time points (Fig. 7c) was scored. − ΔΔCt cutoff ≤ − 0.59 or ≥ + 0.59 (corresponding to 1.5 reduced or increased fold change) coupled with a *p* ≤ 0.05 was selected to include also mild but significant modulations. At 2 days, *HAS1* and *CXCL8* showed upregulation, with *HAS3* and *MMP1-13* being downregulated. Higher *CXCL8* levels could explain the sudden increase of infiltrated neutrophils in synovial fluid treated with MSC-EVs in a porcine model of synovitis [66], being IL-8 one of the most potent chemoattractant molecule. Conversely, at 10 days of EV administration, *CCL2* and *5* and *IL-6* resulted to be significantly downregulated, with *CCL2* and *CCL5* returning at pre-inflammation basal levels. Notably, *CCL5* and *IL-6* are the direct targets of ASC-EV-embedded has-miR17-5p and has-miR106a-5p, respectively. Moreover, *CCL2-5* and *IL-6* are regulated, among others, by NF-kB and MAPK pathways, both target of FLS-unshared ASC-EV miRNAs (MAPK cascade, among DAVID identified Biological Process terms, with *p* = 3.6E−1 and 3 genes). Finally, also *CXCL8* showed a marked contraction, in agreement with its modulation of expression in inflamed FLS by the CREB pathway [67], another postulated target of ASC-EV miRNAs. Finally, to confirm gene expression data, at 10 days release of CCL2, CCL5, and IL-6 were scored by ELISA (Fig. 7d). Notably, values resembled qRT-PCR data, with a 50% reduction of IL-6 levels after prolonged EV administration, CCL2 that returned at basal levels and CCL5 that was detectable only under IL-1β treatment. CCL5 absence of detection in untreated and IL-1β + EV conditions was presumably due to its extremely low amount, in accordance with high qRT-PCR Ct values (> 30). Therefore, these data demonstrated the anti-inflammatory capacity of ASC-EVs in a FLS model of chronic inflammation, suggesting that EV therapy may sustain its efficacy over time also under continuous inflammatory insult, as it may happen in the first days/weeks of treatment.

## Discussion

The use in the orthopedic field of MSC-based products as microfragmented fat or SVF has become a valuable option [68]. Recent literature indicates that in these approaches, MSCs may promote tissue regeneration and reduction of inflammation through paracrine mechanisms relying on both soluble and EV-embedded factors [69], offering exciting therapeutic prospects for the development of off-the-shelf and cell-free biological products [70]. In preliminary in vitro studies assessing the management of OA-affected tissues and cells, ASC-EVs showed promising anti-inflammatory and regenerative features on chondrocytes and osteoblasts [17, 18], confirming their potentiality as a novel therapeutic option [9, 71]. Nevertheless, further data characterizing biogenesis, content, target cell interaction, and potency assays are both needed to shed light on the rationale for EV efficacy and required for manufacture and regulatory oversight, to eventually make ASC-EVs a potential first-in-class new class of therapeutics.

Recent data have indicated that EV content, size, and membrane composition are highly dynamic although the influence of their assembly on the metabolism and structures of the originating cells is still largely undefined, since strongly depending on the heterogeneity of cell types and tissues [72]. In a model of quick starvation, our results showed that EV synthesis and release have a high impact on plasma membrane area and cell volume, confirming the crucial role of EVs as major cell process and not simple waste disposals [73]. In fact, ASC surface reduces from 5.1 × 10^8^ nm^2^ [4*πr*^2^ = 4 × 3.142 × (6.35 × 10^3^ nm)^2^] to 2.4 × 10^8^ nm^2^, with the total surface area of 10,500 EVs released in 24 h being estimated in the order of 3.3 × 10^8^ nm^2^. Similarly, ASCs clear almost 65% of their volume, reducing from 1.1 × 10^12^ nm^3^ to 0.4 × 10^12^ nm^3^, with 0.2 × 10^12^nm^3^ given by EVs. We are aware that these results may be in part influenced by the slowdown of cell metabolism, as reported in a recent work showing that EV secretion in MSCs under starvation gradually reduces over time [16]. However, in view of clinical translation, starvation seems among the most practical method to isolate high yields of very pure EVs. In fact, lipoprotein particles and/or proteins still remain a major potential contaminant from FBS- containing medium, even if depleted of serum-derived EVs [74, 75]. Therefore, waiting for validated synthetic GMP-grade serum-free media, to date, this approach remains the most pragmatic option in view of obtaining clinical products to be readily available.

To date, the majority of studies focused their attention on the protein composition on both EVs and receiver cell surfaces. Available data suggest that EVs can interact with target cells through a ligand-to-receptor synergy and an increasing number of publications confirmed that targeting mediated by specific EV adhesion molecules is among the mechanisms involved in cell binding [76]. In this frame, it was proposed that the isoforms of integrins and surface proteins expressed on EVs may contribute to their delivery to specific organs and tissues, suggesting specific recipient cells’ uptake capabilities [77, 78]. Only recently alternative mechanisms have been investigated involving cooperation between EVs and cell-extracellular matrix. EVs were proposed as one of the structural and functional components of HA-enriched ECM [79], opening the intriguing possibility of a direct bond between EVs and HA. Data herein presented confirmed that HA matrix of synoviocytes is a crucial actor regulating ASC-EV uptake and that such interaction is in part mediated by CD44 levels on vesicle surface. Since CD44 masking on ASC-EVs did not abolish uptake, ECM soaking depending on EV passive diffusion together with the presence of either alternative HA receptors or HA coating of MSC-EVs [80] may also contribute. In fact, CD44 was demonstrated to be expressed also on synoviocytes surface, making HA-EVs and CD44-FLSs interplay an alternative mean of action [81]. Further, exploiting the EV–HA interaction might be an interesting way to improve future ASC-EV therapy in all those cell types and tissues surrounded by a HA matrix, such as articular synoviocytes and chondrocytes. In this perspective, the modulation of CD44 on EVs allowed an increased incorporation. Similarly, soluble HA supplementation resembling physiological synovial fluid environment was able to improve uptake kinetics of ASC-EVs. This, in addition to a reduced Brownian motion at synoviocyte surface increasing docking time, might be due to the proposed role for soluble HA in commuting CD44+ vesicles. To corroborate this hypothesis, CD44 was detected on EVs isolated from both healthy synovial fluid and culture media from chondrocytes or synoviocytes, both enriched in soluble HA [59]. Altogether, these data open two intriguing matters related to future clinical options, such as the role of articular HA concentration and ECM thickness on differential EV diffusion or uptake, that may influence therapeutic choice and potency of administered particles; and the need for the development of future therapeutic products combining soluble HA, already used on for the treatment of OA [82], and ASC-EVs. In this view, a combined therapeutic product, based on HA-EV bond, may also have an impact on ASC-EV biodistribution and therefore efficacy over time after administration.In fact, few but consistent reports showed that, after systemic delivery of EVs, the liver, spleen, and lungs are the preferential target organs, thus spurring different strategies for targeting EVs to specific tissues and enhance their therapeutic efficacy reducing possible off-target effects [83]. Therefore, a combined HA-EV product may be interesting in view of limiting ASC-EV systemic diffusion and maintaining their persistence in the site of action, due to the absence of HA diffusion outside the joint cavity.

In view of clinical applications, a broad characterization of ASC-EV content is mandatory to give a more complete picture of efficacy and also comply with regulatory requirements for advanced medicinal products. A deeper analysis of the miRNA cargo revealed that the shuttled signal is highly conserved among particles isolated from independent ASCs. Although this result adds a crucial brick for the suitability of ASC-EVs as medicinal products also in the allogeneic setting, where a known and conserved array of molecules is mandatory, the role of miRNAs in mediating ASC-EV therapeutic activity has to be critically considered. Recent findings showed that each MSC-EV may carry up to one miRNA and that, in general, around 100 EVs are required to transfer one copy of an abundant miRNA [50, 51]. This implies that when 100,000 ASC-EVs are taken up in 24 h, around 1000 miRNAs in total are engulfed with few to hundreds of copies of the most enriched miRNAs represented, opening crucial issues on their biological significance. In fact, in different cell types, such as cancer cells, liver cells, or hematopoietic stem cells, the total amount of miRNA molecules per cell resulted to be between 10^4^ and 10^5^, with the most represented miRNAs in the 10^2^ to 10^3^ range [84, 85]. Under these premises, and considering that the miRNA–target cell interaction is regulated by a titration mechanism [86], only the most represented miRNAs in EVs that are poorly present in target cells should be considered to predict efficacy, and solely on the subset of transcripts present in receiving cells. In addition, since miRNAs are more stable than mRNAs with half-lives ranging from several hours to days [87], continuous EV uptake and miRNA gathering in target cells suggest that miRNA-dependent EV potency may increase over time even for miRNAs incorporated in few copies per day. Consistently, in inflamed hFLS reduction of chemokine and cytokine mRNA abundance, as *CCL5* and *IL-6*, a direct target of has-miR17-5p and has-miR106a-5p, appeared at 10 days, although overall EV influence on other mechanism or pathways affecting transcription regulation may not be ruled out. Therefore, a clear correlation between transferred miRNAs and temporal modulation of transcript that are not direct targets but under the control of upstream miRNA-regulated pathways is not predictable, and only the general miRNA-dependent effects on the recipient cells may be forecasted. As an example, both *CCL2* and *MMP13* are possible targets of NF-kB, one of the ASC-EV miRNA targets, but their reduction resulted to be temporally distinct although in a pattern of inflammation reduction, as by predicted whole miRNA-dependent effect on inflamed synoviocyte transcriptome. Moreover, due to EV complexity in terms of shuttled molecules, the overall EV-mediated therapeutic effect scored in inflamed synoviocytes has to be considered beyond solely miRNA presence. A critical consideration includes the potential of other EV-embedded players, as proteins or lipids, in a therapeutic dose to elicit a biologically relevant activity. Based on these considerations, cytokines and chemokines could contribute to ASC-EV therapeutic activity observed in both synoviocytes and chondrocytes [18]. In this frame, Alcaraz group nicely demonstrated the presence in ASC-EVs, with particular overrepresentation in microvesicle fraction, of proteins involved in the immune response and particularly of annexin A1 [18], that exerts complex anti-inflammatory and pro-resolution effects together with suppressive capacity on cells of both cartilage [88] and the immune system [89].

## Conclusions

**Fig. 8:**
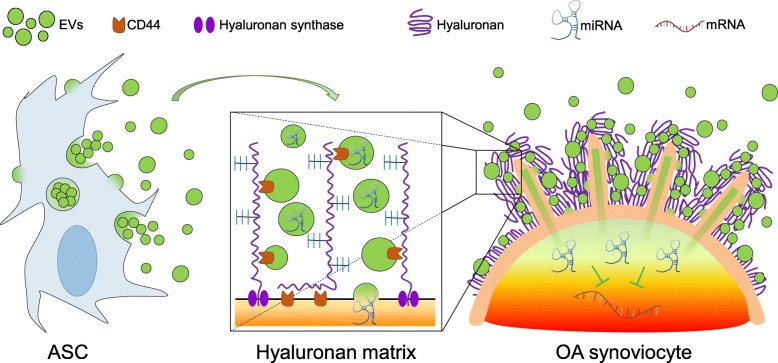
ASC-EVs interact with inflamed synoviocyte hyaluronan matrix enhancing their uptake and eventually release therapeutic miRNAs

This work suggests a new mechanism at the basis of the not fully characterized process of ASC-EV docking and uptake by recipient cells. In fact, the hyaluronan coat acts as a natural sponge for EVs (Fig. 8), complementing the recent findings about the cooperation between EVs and ECM [66]. Moreover, we showed that ASC-EVs have immunoregulatory properties in human OA FLSs even under continuous inflammatory stimuli, resembling what recently observed for OA chondrocytes [18] under acute inflammation. These pioneering results reinforce the great expectations for ASC-EVs as a novel therapeutic agent for osteoarthritis to improve joint homeostasis and prevent a further worsening of joint degeneration. This option would be of great benefit for patients, avoiding or at least postponing the necessity for more invasive procedures such as joint replacement. Nevertheless, deeper analysis of both EV content and their impact on other joint cell and tissues, as immune cells present in synovial fluid, is mandatory to give a more complete picture of efficacy and comply with regulatory requirements for advanced medicinal products in order to introduce ASC-EVs into the clinical practice.

## Additional file

Additional file 1: **Table S1.** miRNA expression in ASC-EVs (not normalized).** Table S2.** Shared miRNAs in ASC-EVs (normalized). **Table S3.** Top 63 ASC-EV-embedded miRNAs falling in the first quartile of expression. **Table S4. **ASC-EVs miRNAs in the first quartile of expression and OA-related [52]. **Table S5.** Targets expressed in IL-1β-treated FLSs for 11 miRNAs present only in ASC-EVs. (DOCX 53 kb)

## Abbreviations

ASCs: Adipose tissue-derived MSCs
CFSE: Carboxy fluorescein diacetate succinimidyl ester
ECM: Extra cellular matrix
EVs: Extracellular vesicles
FLSs: Fibroblast-like synoviocytes
HA: Hyaluronic acid
MSCs: Mesenchymal stromal/stem cells
OA: Osteoarthritis
