# Supplementary material for: Interaction with hyaluronan matrix and miRNA cargo as contributors for in vitro potential of mesenchymal stem cell-derived extracellular vesicles in a model of human osteoarthritic synoviocytes
Source: Stem Cell Res Ther. 2019 Mar 29;10:109. doi: 10.1186/s13287-019-1215-z (PMC6440078; doi:10.1186/s13287-019-1215-z)
Supplement: Supplementary file 1 — Table S1. miRNA expression in ASC-EVs (not normalized). Table S2. Shared miRNAs in ASC-EVs (normalized). Table S3. Top 63 ASC-EV-embedded miRNAs falling in the first quartile of expression. Table S4. ASC-EVs miRNAs in the first quartile of expression and OA-related [52]. Table S5. Targets expressed in IL-1β-treated FLSs for 11 miRNAs present only in ASC-EVs. (DOCX 53 kb) [file 13287_2019_1215_MOESM1_ESM.docx]

Table S1. miRNA expression in ASC-EVs (not normalized)

| ASC1 | C_RT_ | ASC2 | C_RT_ | ASC3 | C_RT_ |
| --- | --- | --- | --- | --- | --- |
| hsa-let-7a_000377 | 14.36 | hsa-let-7a_000377 | 15.65 | hsa-let-7a_000377 | 18.19 |
| hsa-let-7b_002619 | 13.82 | hsa-let-7b_002619 | 14.59 | hsa-let-7c_000379 | 16.78 |
| hsa-let-7c_000379 | 15.84 | hsa-let-7c_000379 | 15.73 | hsa-let-7d_002283 | 21.71 |
| hsa-let-7d_002283 | 18.59 | hsa-let-7d_002283 | 19.80 | hsa-let-7g_002282 | 19.46 |
| hsa-let-7e_002406 | 14.15 | hsa-let-7e_002406 | 15.73 | hsa-miR-100_000437 | 12.58 |
| hsa-let-7f_000382 | 17.52 | hsa-let-7f_000382 | 18.51 | hsa-miR-101_002253 | 23.32 |
| hsa-let-7f-2#_002418 | 26.80 | hsa-let-7g_002282 | 18.40 | hsa-miR-103_000439 | 20.08 |
| hsa-let-7g_002282 | 17.83 | hsa-miR-100_000437 | 11.94 | hsa-miR-106a_002169 | 14.75 |
| hsa-let-7i#_002172 | 26.76 | hsa-miR-101_002253 | 22.76 | hsa-miR-106b_000442 | 15.36 |
| hsa-miR-100#_002142 | 26.52 | hsa-miR-103_000439 | 18.96 | hsa-miR-10a#_002288 | 24.72 |
| hsa-miR-100_000437 | 10.88 | hsa-miR-106a_002169 | 15.81 | hsa-miR-10a_000387 | 16.84 |
| hsa-miR-101_002253 | 21.62 | hsa-miR-106b#_002380 | 20.22 | hsa-miR-10b#_002315 | 17.93 |
| hsa-miR-103_000439 | 16.85 | hsa-miR-106b_000442 | 15.63 | hsa-miR-10b_002218 | 19.72 |
| hsa-miR-106a_002169 | 14.34 | hsa-miR-10a#_002288 | 25.76 | hsa-miR-1180_002847 | 22.31 |
| hsa-miR-106b_000442 | 14.70 | hsa-miR-10a_000387 | 15.41 | hsa-miR-1226#_002758 | 21.88 |
| hsa-miR-107_000443 | 22.58 | hsa-miR-10b#_002315 | 18.08 | hsa-miR-1244_002791 | 26.93 |
| hsa-miR-10a#_002288 | 24.77 | hsa-miR-10b_002218 | 18.04 | hsa-miR-1255B_002801 | 22.22 |
| hsa-miR-10a_000387 | 14.79 | hsa-miR-1180_002847 | 22.50 | hsa-miR-125a-3p_002199 | 26.37 |
| hsa-miR-10b#_002315 | 17.64 | hsa-miR-1226#_002758 | 22.92 | hsa-miR-125a-5p_002198 | 18.96 |
| hsa-miR-10b_002218 | 16.90 | hsa-miR-1236_002761 | 23.88 | hsa-miR-125b_000449 | 10.32 |
| hsa-miR-1180_002847 | 23.16 | hsa-miR-1254_002818 | 26.81 | hsa-miR-125b-1#_002378 | 20.81 |
| hsa-miR-1226#_002758 | 23.01 | hsa-miR-1255B_002801 | 24.44 | hsa-miR-125b-2#_002158 | 22.83 |
| hsa-miR-1227_002769 | 22.81 | hsa-miR-125a-3p_002199 | 26.51 | hsa-miR-126#_000451 | 25.76 |
| hsa-miR-1244_002791 | 25.60 | hsa-miR-125a-5p_002198 | 18.50 | hsa-miR-126_002228 | 25.09 |
| hsa-miR-1255B_002801 | 22.83 | hsa-miR-125b_000449 | 9.82 | hsa-miR-1260_002896 | 16.37 |
| hsa-miR-125a-3p_002199 | 24.90 | hsa-miR-125b-1#_002378 | 21.45 | hsa-miR-127_000452 | 14.76 |
| hsa-miR-125a-5p_002198 | 17.73 | hsa-miR-126#_000451 | 25.77 | hsa-miR-1270_002807 | 25.83 |
| hsa-miR-125b_000449 | 8.80 | hsa-miR-126_002228 | 24.42 | hsa-miR-1271_002779 | 20.81 |
| hsa-miR-125b-1#_002378 | 20.51 | hsa-miR-1260_002896 | 15.77 | hsa-miR-1274A_002883 | 9.66 |
| hsa-miR-126#_000451 | 23.57 | hsa-miR-127_000452 | 14.03 | hsa-miR-1274B_002884 | 7.84 |
| hsa-miR-126_002228 | 21.42 | hsa-miR-1270_002807 | 26.41 | hsa-miR-1276_002843 | 25.17 |
| hsa-miR-1260_002896 | 16.54 | hsa-miR-1271_002779 | 22.21 | hsa-miR-1285_002822 | 21.62 |
| hsa-miR-1265_002790 | 26.73 | hsa-miR-1274A_002883 | 9.73 | hsa-miR-128a_002216 | 20.58 |
| hsa-miR-127_000452 | 14.39 | hsa-miR-1274B_002884 | 7.75 | hsa-miR-1290_002863 | 21.46 |
| hsa-miR-1270_002807 | 25.87 | hsa-miR-1285_002822 | 24.66 | hsa-miR-1291_002838 | 17.93 |
| hsa-miR-1271_002779 | 22.33 | hsa-miR-128a_002216 | 20.78 | hsa-miR-1296_002908 | 19.06 |
| hsa-miR-1274A_002883 | 10.82 | hsa-miR-1290_002863 | 21.49 | hsa-miR-1300_002902 | 25.22 |
| hsa-miR-1274B_002884 | 8.79 | hsa-miR-1291_002838 | 17.75 | hsa-miR-1303_002792 | 21.58 |
| hsa-miR-1276_002843 | 24.34 | hsa-miR-1296_002908 | 19.93 | hsa-miR-130a_000454 | 14.61 |
| hsa-miR-1285_002822 | 22.51 | hsa-miR-1300_002902 | 25.36 | hsa-miR-130b_000456 | 17.41 |
| hsa-miR-128a_002216 | 20.13 | hsa-miR-1303_002792 | 22.91 | hsa-miR-132_000457 | 15.46 |
| hsa-miR-129#_002298 | 26.22 | hsa-miR-130a_000454 | 14.63 | hsa-miR-135b_002261 | 26.80 |
| hsa-miR-1290_002863 | 21.81 | hsa-miR-130b_000456 | 17.62 | hsa-miR-136#_002100 | 21.97 |
| hsa-miR-1291_002838 | 17.11 | hsa-miR-132_000457 | 14.92 | hsa-miR-138_002284 | 16.78 |
| hsa-miR-1300_002902 | 24.06 | hsa-miR-133a_002246 | 24.09 | hsa-miR-139-5p_002289 | 23.14 |
| hsa-miR-1303_002792 | 21.60 | hsa-miR-136#_002100 | 22.16 | hsa-miR-140-3p_002234 | 22.60 |
| hsa-miR-130a_000454 | 13.69 | hsa-miR-138_002284 | 16.53 | hsa-miR-143_002249 | 17.84 |
| hsa-miR-130b#_002114 | 24.48 | hsa-miR-139-5p_002289 | 22.27 | hsa-miR-145#_002149 | 24.34 |
| hsa-miR-130b_000456 | 16.96 | hsa-miR-140-3p_002234 | 22.24 | hsa-miR-145_002278 | 13.78 |
| hsa-miR-132_000457 | 13.74 | hsa-miR-142-3p_000464 | 25.81 | hsa-miR-146a_000468 | 21.92 |
| hsa-miR-135b_002261 | 25.02 | hsa-miR-143_002249 | 16.72 | hsa-miR-146b_001097 | 19.36 |
| hsa-miR-136#_002100 | 22.24 | hsa-miR-145#_002149 | 23.51 | hsa-miR-146b-3p_002361 | 25.21 |
| hsa-miR-138_002284 | 14.82 | hsa-miR-145_002278 | 12.80 | hsa-miR-148a_000470 | 17.31 |
| hsa-miR-139-5p_002289 | 21.85 | hsa-miR-146a_000468 | 24.02 | hsa-miR-148b#_002160 | 24.84 |
| hsa-miR-140-3p_002234 | 20.96 | hsa-miR-146b_001097 | 18.88 | hsa-miR-148b_000471 | 21.78 |
| hsa-miR-142-3p_000464 | 25.83 | hsa-miR-146b-3p_002361 | 25.24 | hsa-miR-149_002255 | 17.15 |
| hsa-miR-143_002249 | 15.91 | hsa-miR-148a_000470 | 17.51 | hsa-miR-150_000473 | 23.27 |
| hsa-miR-145#_002149 | 23.22 | hsa-miR-148b#_002160 | 25.75 | hsa-miR-151-3p_002254 | 18.85 |
| hsa-miR-145_002278 | 12.27 | hsa-miR-148b_000471 | 21.59 | hsa-miR-151-5P_002642 | 23.08 |
| hsa-miR-146a_000468 | 19.69 | hsa-miR-149_002255 | 16.73 | hsa-miR-152_000475 | 15.07 |
| hsa-miR-146b_001097 | 17.81 | hsa-miR-150_000473 | 24.83 | hsa-miR-154#_000478 | 24.45 |
| hsa-miR-146b-3p_002361 | 24.07 | hsa-miR-151-3p_002254 | 19.81 | hsa-miR-154_000477 | 22.21 |
| hsa-miR-148a_000470 | 16.78 | hsa-miR-151-5P_002642 | 22.67 | hsa-miR-155_002623 | 22.20 |
| hsa-miR-148b#_002160 | 24.59 | hsa-miR-152_000475 | 14.65 | hsa-miR-15a#_002419 | 25.04 |
| hsa-miR-148b_000471 | 20.40 | hsa-miR-154#_000478 | 24.27 | hsa-miR-15a_000389 | 22.14 |
| hsa-miR-149_002255 | 16.59 | hsa-miR-154_000477 | 21.70 | hsa-miR-15b_000390 | 17.43 |
| hsa-miR-150_000473 | 21.76 | hsa-miR-155_002623 | 22.18 | hsa-miR-16_000391 | 15.29 |
| hsa-miR-151-3p_002254 | 17.86 | hsa-miR-15a#_002419 | 26.93 | hsa-miR-16-1#_002420 | 25.85 |
| hsa-miR-151-5P_002642 | 21.18 | hsa-miR-15a_000389 | 21.42 | hsa-miR-17_002308 | 14.77 |
| hsa-miR-152_000475 | 14.02 | hsa-miR-15b_000390 | 16.88 | hsa-miR-181a_000480 | 18.67 |
| hsa-miR-154#_000478 | 23.64 | hsa-miR-16_000391 | 15.02 | hsa-miR-181a-2#_002317 | 24.63 |
| hsa-miR-154_000477 | 21.20 | hsa-miR-16-1#_002420 | 26.38 | hsa-miR-181c_000482 | 23.81 |
| hsa-miR-155_002623 | 20.47 | hsa-miR-17_002308 | 15.47 | hsa-miR-184_000485 | 22.77 |
| hsa-miR-15a#_002419 | 24.31 | hsa-miR-181a_000480 | 16.96 | hsa-miR-185_002271 | 20.86 |
| hsa-miR-15a_000389 | 19.60 | hsa-miR-181a-2#_002317 | 23.92 | hsa-miR-186_002285 | 18.86 |
| hsa-miR-15b_000390 | 15.97 | hsa-miR-181c_000482 | 23.25 | hsa-miR-18a#_002423 | 25.83 |
| hsa-miR-16_000391 | 14.18 | hsa-miR-184_000485 | 21.31 | hsa-miR-18a_002422 | 20.83 |
| hsa-miR-16-1#_002420 | 25.19 | hsa-miR-185_002271 | 21.18 | hsa-miR-190_000489 | 23.69 |
| hsa-miR-17_002308 | 14.24 | hsa-miR-186_002285 | 18.99 | hsa-miR-190b_002263 | 26.83 |
| hsa-miR-181a_000480 | 15.05 | hsa-miR-18a#_002423 | 26.54 | hsa-miR-191_002299 | 13.27 |
| hsa-miR-181a-2#_002317 | 21.95 | hsa-miR-18a_002422 | 21.75 | hsa-miR-192_000491 | 20.55 |
| hsa-miR-181c_000482 | 20.92 | hsa-miR-190_000489 | 21.83 | hsa-miR-193a-3p_002250 | 26.41 |
| hsa-miR-184_000485 | 20.66 | hsa-miR-191_002299 | 13.20 | hsa-miR-193a-5p_002281 | 16.53 |
| hsa-miR-185_002271 | 20.49 | hsa-miR-192_000491 | 20.78 | hsa-miR-193b#_002366 | 20.36 |
| hsa-miR-186_002285 | 17.75 | hsa-miR-193a-5p_002281 | 16.59 | hsa-miR-193b_002367 | 11.80 |
| hsa-miR-18a#_002423 | 26.48 | hsa-miR-193b#_002366 | 20.54 | hsa-miR-194_000493 | 24.42 |
| hsa-miR-18a_002422 | 21.11 | hsa-miR-193b_002367 | 11.78 | hsa-miR-195_000494 | 17.22 |
| hsa-miR-190_000489 | 20.83 | hsa-miR-194_000493 | 24.08 | hsa-miR-196b_002215 | 24.73 |
| hsa-miR-190b_002263 | 25.70 | hsa-miR-195_000494 | 16.69 | hsa-miR-197_000497 | 15.26 |
| hsa-miR-191_002299 | 12.50 | hsa-miR-196b_002215 | 22.92 | hsa-miR-198_002273 | 25.72 |
| hsa-miR-192_000491 | 19.28 | hsa-miR-197_000497 | 14.78 | hsa-miR-199a_000498 | 19.49 |
| hsa-miR-193a-5p_002281 | 15.77 | hsa-miR-198_002273 | 23.18 | hsa-miR-199a-3p_002304 | 14.40 |
| hsa-miR-193b#_002366 | 19.88 | hsa-miR-199a_000498 | 18.84 | hsa-miR-199b_000500 | 18.80 |
| hsa-miR-193b_002367 | 11.09 | hsa-miR-199a-3p_002304 | 13.66 | hsa-miR-19a_000395 | 18.68 |
| hsa-miR-194_000493 | 21.36 | hsa-miR-199b_000500 | 18.40 | hsa-miR-19b_000396 | 13.38 |
| hsa-miR-195_000494 | 16.09 | hsa-miR-19a_000395 | 19.10 | hsa-miR-19b-1#_002425 | 25.12 |
| hsa-miR-196b_002215 | 22.08 | hsa-miR-19b_000396 | 13.73 | hsa-miR-203_000507 | 22.88 |
| hsa-miR-197_000497 | 14.71 | hsa-miR-19b-1#_002425 | 25.97 | hsa-miR-204_000508 | 20.21 |
| hsa-miR-198_002273 | 23.37 | hsa-miR-203_000507 | 23.40 | hsa-miR-206_000510 | 26.50 |
| hsa-miR-199a_000498 | 18.03 | hsa-miR-204_000508 | 17.72 | hsa-miR-20a#_002437 | 26.89 |
| hsa-miR-199a-3p_002304 | 13.35 | hsa-miR-206_000510 | 26.83 | hsa-miR-20a_000580 | 13.13 |
| hsa-miR-199b_000500 | 18.51 | hsa-miR-20a#_002437 | 26.75 | hsa-miR-20b_001014 | 21.27 |
| hsa-miR-19a_000395 | 18.46 | hsa-miR-20a_000580 | 13.65 | hsa-miR-21_000397 | 11.68 |
| hsa-miR-19b_000396 | 13.06 | hsa-miR-20b_001014 | 23.57 | hsa-miR-210_000512 | 16.50 |
| hsa-miR-19b-1#_002425 | 25.12 | hsa-miR-21_000397 | 11.20 | hsa-miR-212_000515 | 19.43 |
| hsa-miR-203_000507 | 21.79 | hsa-miR-210_000512 | 16.61 | hsa-miR-214#_002293 | 21.18 |
| hsa-miR-204_000508 | 17.78 | hsa-miR-212_000515 | 19.23 | hsa-miR-214_002306 | 14.22 |
| hsa-miR-206_000510 | 25.74 | hsa-miR-214#_002293 | 21.57 | hsa-miR-218_000521 | 13.81 |
| hsa-miR-20a#_002437 | 25.27 | hsa-miR-214_002306 | 13.77 | hsa-miR-218-2#_002294 | 26.72 |
| hsa-miR-20a_000580 | 12.59 | hsa-miR-218_000521 | 14.35 | hsa-miR-22#_002301 | 19.03 |
| hsa-miR-20b_001014 | 21.63 | hsa-miR-218-2#_002294 | 26.42 | hsa-miR-22_000398 | 16.69 |
| hsa-miR-21_000397 | 10.12 | hsa-miR-219_000522 | 26.69 | hsa-miR-221#_002096 | 25.76 |
| hsa-miR-210_000512 | 15.81 | hsa-miR-22#_002301 | 18.81 | hsa-miR-221_000524 | 10.78 |
| hsa-miR-212_000515 | 17.82 | hsa-miR-22_000398 | 16.55 | hsa-miR-222#_002097 | 19.24 |
| hsa-miR-213_000516 | 22.43 | hsa-miR-221#_002096 | 25.51 | hsa-miR-222_002276 | 11.81 |
| hsa-miR-214#_002293 | 20.33 | hsa-miR-221_000524 | 11.71 | hsa-miR-223_002295 | 24.52 |
| hsa-miR-214_002306 | 13.31 | hsa-miR-222#_002097 | 20.06 | hsa-miR-224_002099 | 14.76 |
| hsa-miR-218_000521 | 13.95 | hsa-miR-222_002276 | 11.42 | hsa-miR-23a#_002439 | 25.03 |
| hsa-miR-219_000522 | 26.37 | hsa-miR-223_002295 | 24.33 | hsa-miR-23a_000399 | 17.42 |
| hsa-miR-22#_002301 | 18.53 | hsa-miR-224_002099 | 14.44 | hsa-miR-24_000402 | 10.03 |
| hsa-miR-22_000398 | 15.69 | hsa-miR-23a#_002439 | 26.14 | hsa-miR-24-2#_002441 | 20.75 |
| hsa-miR-221#_002096 | 23.73 | hsa-miR-23a_000399 | 17.09 | hsa-miR-25_000403 | 15.76 |
| hsa-miR-221_000524 | 10.81 | hsa-miR-24_000402 | 9.67 | hsa-miR-26a_000405 | 15.50 |
| hsa-miR-222#_002097 | 19.51 | hsa-miR-24-2#_002441 | 21.00 | hsa-miR-26b_000407 | 17.29 |
| hsa-miR-222_002276 | 10.59 | hsa-miR-25_000403 | 16.47 | hsa-miR-27a#_002445 | 22.84 |
| hsa-miR-223_002295 | 22.76 | hsa-miR-26a_000405 | 14.30 | hsa-miR-27a_000408 | 14.17 |
| hsa-miR-224_002099 | 14.40 | hsa-miR-26b_000407 | 16.40 | hsa-miR-27b#_002174 | 24.31 |
| hsa-miR-23a#_002439 | 24.68 | hsa-miR-27a#_002445 | 23.15 | hsa-miR-27b_000409 | 15.59 |
| hsa-miR-23a_000399 | 16.27 | hsa-miR-27a_000408 | 13.65 | hsa-miR-28_000411 | 17.30 |
| hsa-miR-24_000402 | 8.94 | hsa-miR-27b#_002174 | 22.86 | hsa-miR-28-3p_002446 | 17.20 |
| hsa-miR-24-2#_002441 | 19.83 | hsa-miR-27b_000409 | 14.94 | hsa-miR-296_000527 | 16.48 |
| hsa-miR-25_000403 | 15.78 | hsa-miR-28_000411 | 16.73 | hsa-miR-296-3p_002101 | 24.03 |
| hsa-miR-26a_000405 | 13.62 | hsa-miR-28-3p_002446 | 17.09 | hsa-miR-299-5p_000600 | 23.92 |
| hsa-miR-26b_000407 | 15.75 | hsa-miR-296_000527 | 16.16 | hsa-miR-29a#_002447 | 17.81 |
| hsa-miR-27a#_002445 | 22.75 | hsa-miR-296-3p_002101 | 24.33 | hsa-miR-29a_002112 | 14.08 |
| hsa-miR-27a_000408 | 13.24 | hsa-miR-299-5p_000600 | 23.31 | hsa-miR-29b_000413 | 17.89 |
| hsa-miR-27b#_002174 | 23.58 | hsa-miR-29a#_002447 | 17.90 | hsa-miR-29c_000587 | 15.43 |
| hsa-miR-27b_000409 | 14.90 | hsa-miR-29a_002112 | 14.16 | hsa-miR-301_000528 | 19.30 |
| hsa-miR-28_000411 | 15.56 | hsa-miR-29b_000413 | 17.66 | hsa-miR-301b_002392 | 24.01 |
| hsa-miR-28-3p_002446 | 16.46 | hsa-miR-29c_000587 | 15.30 | hsa-miR-302a_000529 | 22.77 |
| hsa-miR-296_000527 | 16.14 | hsa-miR-301_000528 | 19.32 | hsa-miR-30a-3p_000416 | 17.39 |
| hsa-miR-296-3p_002101 | 23.91 | hsa-miR-301b_002392 | 23.82 | hsa-miR-30a-5p_000417 | 14.66 |
| hsa-miR-299-5p_000600 | 23.06 | hsa-miR-30a-3p_000416 | 17.11 | hsa-miR-30b_000602 | 12.87 |
| hsa-miR-29a#_002447 | 17.55 | hsa-miR-30a-5p_000417 | 14.53 | hsa-miR-30c_000419 | 12.52 |
| hsa-miR-29a_002112 | 12.80 | hsa-miR-30b_000602 | 12.60 | hsa-miR-30d#_002305 | 25.25 |
| hsa-miR-29b_000413 | 15.82 | hsa-miR-30c_000419 | 12.19 | hsa-miR-30d_000420 | 17.14 |
| hsa-miR-29b-2#_002166 | 26.64 | hsa-miR-30d#_002305 | 25.99 | hsa-miR-30e-3p_000422 | 17.23 |
| hsa-miR-29c_000587 | 13.81 | hsa-miR-30d_000420 | 16.86 | hsa-miR-31#_002113 | 15.56 |
| hsa-miR-301_000528 | 18.66 | hsa-miR-30e-3p_000422 | 16.79 | hsa-miR-31_002279 | 14.44 |
| hsa-miR-301b_002392 | 22.90 | hsa-miR-31#_002113 | 15.72 | hsa-miR-32_002109 | 26.25 |
| hsa-miR-302a_000529 | 18.05 | hsa-miR-31_002279 | 13.69 | hsa-miR-320_002277 | 15.29 |
| hsa-miR-302b_000531 | 26.78 | hsa-miR-32_002109 | 26.79 | hsa-miR-320B_002844 | 19.72 |
| hsa-miR-302d_000535 | 26.75 | hsa-miR-320_002277 | 14.99 | hsa-miR-323-3p_002227 | 20.81 |
| hsa-miR-30a-3p_000416 | 16.57 | hsa-miR-320B_002844 | 19.95 | hsa-miR-324-3p_002161 | 20.55 |
| hsa-miR-30a-5p_000417 | 13.62 | hsa-miR-323-3p_002227 | 21.23 | hsa-miR-324-5p_000539 | 18.15 |
| hsa-miR-30b_000602 | 11.80 | hsa-miR-324-3p_002161 | 20.52 | hsa-miR-328_000543 | 14.83 |
| hsa-miR-30c_000419 | 11.41 | hsa-miR-324-5p_000539 | 17.72 | hsa-miR-329_001101 | 23.28 |
| hsa-miR-30d#_002305 | 24.82 | hsa-miR-328_000543 | 14.48 | hsa-miR-330_000544 | 21.07 |
| hsa-miR-30d_000420 | 16.35 | hsa-miR-329_001101 | 22.80 | hsa-miR-331_000545 | 16.41 |
| hsa-miR-30e-3p_000422 | 16.43 | hsa-miR-330_000544 | 21.39 | hsa-miR-335_000546 | 17.72 |
| hsa-miR-31#_002113 | 14.40 | hsa-miR-331_000545 | 15.39 | hsa-miR-337-3p_002157 | 23.70 |
| hsa-miR-31_002279 | 11.65 | hsa-miR-331-5p_002233 | 23.10 | hsa-miR-337-5p_002156 | 21.56 |
| hsa-miR-32_002109 | 25.76 | hsa-miR-335#_002185 | 22.64 | hsa-miR-338-5P_002658 | 25.43 |
| hsa-miR-320_002277 | 14.21 | hsa-miR-335_000546 | 17.76 | hsa-miR-339-3p_002184 | 21.82 |
| hsa-miR-320B_002844 | 19.44 | hsa-miR-337-3p_002157 | 24.26 | hsa-miR-33a#_002136 | 26.70 |
| hsa-miR-323-3p_002227 | 21.72 | hsa-miR-337-5p_002156 | 21.64 | hsa-miR-340#_002259 | 26.59 |
| hsa-miR-324-3p_002161 | 19.46 | hsa-miR-339-3p_002184 | 21.74 | hsa-miR-340_002258 | 25.10 |
| hsa-miR-324-5p_000539 | 16.41 | hsa-miR-339-5p_002257 | 17.62 | hsa-miR-342-3p_002260 | 18.41 |
| hsa-miR-328_000543 | 14.10 | hsa-miR-33a#_002136 | 26.79 | hsa-miR-345_002186 | 21.04 |
| hsa-miR-329_001101 | 23.34 | hsa-miR-340#_002259 | 26.76 | hsa-miR-34a#_002316 | 17.44 |
| hsa-miR-330_000544 | 19.82 | hsa-miR-340_002258 | 24.30 | hsa-miR-34a_000426 | 14.80 |
| hsa-miR-331_000545 | 14.66 | hsa-miR-342-3p_002260 | 18.00 | hsa-miR-34b_000427 | 22.36 |
| hsa-miR-335#_002185 | 21.25 | hsa-miR-345_002186 | 20.66 | hsa-miR-34b_002102 | 18.28 |
| hsa-miR-335_000546 | 15.72 | hsa-miR-34a#_002316 | 17.34 | hsa-miR-34c_000428 | 18.53 |
| hsa-miR-337-3p_002157 | 24.29 | hsa-miR-34a_000426 | 14.10 | hsa-miR-361_000554 | 17.32 |
| hsa-miR-337-5p_002156 | 21.82 | hsa-miR-34b_000427 | 22.45 | hsa-miR-362_001273 | 24.49 |
| hsa-miR-338-5P_002658 | 24.82 | hsa-miR-34b_002102 | 18.72 | hsa-miR-362-3p_002117 | 23.56 |
| hsa-miR-339-3p_002184 | 21.23 | hsa-miR-34c_000428 | 18.10 | hsa-miR-365_001020 | 16.54 |
| hsa-miR-339-5p_002257 | 17.10 | hsa-miR-361_000554 | 17.31 | hsa-miR-369-3p_000557 | 23.44 |
| hsa-miR-33a#_002136 | 25.47 | hsa-miR-362_001273 | 23.67 | hsa-miR-369-5p_001021 | 26.84 |
| hsa-miR-340#_002259 | 24.45 | hsa-miR-362-3p_002117 | 23.79 | hsa-miR-370_002275 | 17.34 |
| hsa-miR-340_002258 | 23.78 | hsa-miR-365_001020 | 16.61 | hsa-miR-372_000560 | 24.82 |
| hsa-miR-342-3p_002260 | 17.77 | hsa-miR-369-3p_000557 | 22.47 | hsa-miR-373_000561 | 24.11 |
| hsa-miR-345_002186 | 19.89 | hsa-miR-370_002275 | 17.12 | hsa-miR-374_000563 | 18.97 |
| hsa-miR-34a#_002316 | 16.95 | hsa-miR-372_000560 | 26.73 | hsa-miR-375_000564 | 25.78 |
| hsa-miR-34a_000426 | 13.66 | hsa-miR-373_000561 | 24.56 | hsa-miR-376a_000565 | 15.80 |
| hsa-miR-34b_000427 | 21.73 | hsa-miR-374_000563 | 17.96 | hsa-miR-376b_001102 | 24.05 |
| hsa-miR-34b_002102 | 18.13 | hsa-miR-375_000564 | 25.00 | hsa-miR-376c_002122 | 16.49 |
| hsa-miR-34c_000428 | 17.70 | hsa-miR-376a_000565 | 15.84 | hsa-miR-377#_002128 | 25.65 |
| hsa-miR-361_000554 | 16.79 | hsa-miR-376b_001102 | 23.50 | hsa-miR-377_000566 | 25.00 |
| hsa-miR-362_001273 | 21.82 | hsa-miR-376c_002122 | 16.54 | hsa-miR-378_000567 | 25.04 |
| hsa-miR-362-3p_002117 | 23.39 | hsa-miR-377#_002128 | 25.91 | hsa-miR-378_002243 | 22.76 |
| hsa-miR-365_001020 | 15.22 | hsa-miR-378_000567 | 26.74 | hsa-miR-380-3p_000569 | 25.24 |
| hsa-miR-367_000555 | 18.64 | hsa-miR-378_002243 | 23.79 | hsa-miR-380-5p_000570 | 23.55 |
| hsa-miR-369-3p_000557 | 23.38 | hsa-miR-380-5p_000570 | 23.80 | hsa-miR-381_000571 | 21.99 |
| hsa-miR-369-5p_001021 | 26.40 | hsa-miR-381_000571 | 22.41 | hsa-miR-382_000572 | 14.55 |
| hsa-miR-372_000560 | 24.26 | hsa-miR-382_000572 | 13.90 | hsa-miR-383_000573 | 22.79 |
| hsa-miR-373_000561 | 24.59 | hsa-miR-383_000573 | 21.62 | hsa-miR-409-3p_002332 | 15.68 |
| hsa-miR-374_000563 | 16.81 | hsa-miR-409-3p_002332 | 15.14 | hsa-miR-409-5p_002331 | 24.22 |
| hsa-miR-375_000564 | 25.54 | hsa-miR-409-5p_002331 | 23.35 | hsa-miR-410_001274 | 19.35 |
| hsa-miR-376a_000565 | 16.35 | hsa-miR-410_001274 | 18.84 | hsa-miR-411_001610 | 19.35 |
| hsa-miR-376b_001102 | 24.06 | hsa-miR-411_001610 | 18.24 | hsa-miR-423-5p_002340 | 18.83 |
| hsa-miR-376c_002122 | 16.36 | hsa-miR-422a_002297 | 26.93 | hsa-miR-424#_002309 | 19.38 |
| hsa-miR-377#_002128 | 25.01 | hsa-miR-423-5p_002340 | 18.89 | hsa-miR-424_000604 | 19.71 |
| hsa-miR-378_000567 | 24.81 | hsa-miR-424#_002309 | 19.49 | hsa-miR-425#_002302 | 24.43 |
| hsa-miR-378_002243 | 23.64 | hsa-miR-424_000604 | 18.26 | hsa-miR-432_001026 | 21.46 |
| hsa-miR-380-5p_000570 | 24.81 | hsa-miR-425#_002302 | 24.75 | hsa-miR-433_001028 | 22.23 |
| hsa-miR-381_000571 | 22.56 | hsa-miR-432_001026 | 20.90 | hsa-miR-450a_002303 | 25.61 |
| hsa-miR-382_000572 | 14.77 | hsa-miR-433_001028 | 21.58 | hsa-miR-452_002329 | 20.76 |
| hsa-miR-409-3p_002332 | 15.64 | hsa-miR-450a_002303 | 24.86 | hsa-miR-454_002323 | 21.67 |
| hsa-miR-409-5p_002331 | 22.51 | hsa-miR-452_002329 | 20.17 | hsa-miR-455_001280 | 21.73 |
| hsa-miR-410_001274 | 18.62 | hsa-miR-454_002323 | 23.72 | hsa-miR-455-3p_002244 | 21.67 |
| hsa-miR-411_001610 | 17.24 | hsa-miR-455_001280 | 22.19 | hsa-miR-483-5p_002338 | 17.43 |
| hsa-miR-422a_002297 | 26.05 | hsa-miR-455-3p_002244 | 20.95 | hsa-miR-484_001821 | 15.27 |
| hsa-miR-423-5p_002340 | 18.59 | hsa-miR-483-5p_002338 | 16.76 | hsa-miR-485-3p_001277 | 21.69 |
| hsa-miR-424#_002309 | 18.83 | hsa-miR-484_001821 | 15.15 | hsa-miR-487a_001279 | 24.52 |
| hsa-miR-424_000604 | 17.58 | hsa-miR-485-3p_001277 | 20.93 | hsa-miR-487b_001285 | 21.67 |
| hsa-miR-425#_002302 | 23.96 | hsa-miR-487a_001279 | 24.87 | hsa-miR-488_001106 | 23.99 |
| hsa-miR-432_001026 | 22.13 | hsa-miR-487b_001285 | 21.44 | hsa-miR-493_002364 | 23.41 |
| hsa-miR-433_001028 | 21.76 | hsa-miR-493_002364 | 22.82 | hsa-miR-494_002365 | 19.46 |
| hsa-miR-450a_002303 | 23.96 | hsa-miR-494_002365 | 19.28 | hsa-miR-497_001043 | 21.31 |
| hsa-miR-450b-5p_002207 | 26.53 | hsa-miR-497_001043 | 22.04 | hsa-miR-500_001046 | 24.01 |
| hsa-miR-452_002329 | 19.75 | hsa-miR-500_002428 | 22.55 | hsa-miR-500_002428 | 22.82 |
| hsa-miR-454_002323 | 21.57 | hsa-miR-501-3p_002435 | 22.77 | hsa-miR-502_001109 | 23.79 |
| hsa-miR-455_001280 | 20.44 | hsa-miR-502_001109 | 23.21 | hsa-miR-502-3p_002083 | 21.95 |
| hsa-miR-455-3p_002244 | 18.99 | hsa-miR-502-3p_002083 | 22.53 | hsa-miR-503_001048 | 19.98 |
| hsa-miR-483-5p_002338 | 17.99 | hsa-miR-503_001048 | 19.71 | hsa-miR-505#_002087 | 24.74 |
| hsa-miR-484_001821 | 14.35 | hsa-miR-505#_002087 | 24.57 | hsa-miR-505_002089 | 21.82 |
| hsa-miR-485-3p_001277 | 21.77 | hsa-miR-505_002089 | 22.76 | hsa-miR-509-5p_002235 | 24.53 |
| hsa-miR-487a_001279 | 24.47 | hsa-miR-532_001518 | 17.70 | hsa-miR-520D-3P_002743 | 25.80 |
| hsa-miR-487b_001285 | 21.76 | hsa-miR-532-3p_002355 | 19.53 | hsa-miR-526b_002382 | 25.80 |
| hsa-miR-488_001106 | 24.00 | hsa-miR-539_001286 | 21.57 | hsa-miR-532_001518 | 17.63 |
| hsa-miR-489_002358 | 25.69 | hsa-miR-542-3p_001284 | 22.41 | hsa-miR-532-3p_002355 | 19.60 |
| hsa-miR-493_002364 | 22.75 | hsa-miR-542-5p_002240 | 24.77 | hsa-miR-539_001286 | 22.05 |
| hsa-miR-494_002365 | 18.96 | hsa-miR-543_002376 | 20.64 | hsa-miR-542-3p_001284 | 23.11 |
| hsa-miR-497_001043 | 21.46 | hsa-miR-548a_001538 | 26.35 | hsa-miR-542-5p_002240 | 24.62 |
| hsa-miR-500_001046 | 24.29 | hsa-miR-548b-5p_002408 | 24.20 | hsa-miR-543_002376 | 20.48 |
| hsa-miR-500_002428 | 20.77 | hsa-miR-548c-5p_002429 | 24.56 | hsa-miR-545_002267 | 26.37 |
| hsa-miR-501-3p_002435 | 21.83 | hsa-miR-548d_001605 | 26.77 | hsa-miR-548b-5p_002408 | 22.98 |
| hsa-miR-502_001109 | 21.81 | hsa-miR-548d-5p_002237 | 24.95 | hsa-miR-548c-5p_002429 | 23.55 |
| hsa-miR-502-3p_002083 | 21.68 | hsa-miR-572_001614 | 22.48 | hsa-miR-548d-5p_002237 | 23.90 |
| hsa-miR-503_001048 | 19.74 | hsa-miR-574-3p_002349 | 13.90 | hsa-miR-572_001614 | 22.17 |
| hsa-miR-505#_002087 | 23.67 | hsa-miR-576-3p_002351 | 23.07 | hsa-miR-574-3p_002349 | 14.19 |
| hsa-miR-505_002089 | 21.38 | hsa-miR-579_002398 | 23.80 | hsa-miR-576-3p_002351 | 22.81 |
| hsa-miR-509-5p_002235 | 22.43 | hsa-miR-589_001543 | 23.05 | hsa-miR-579_002398 | 23.40 |
| hsa-miR-516-3p_001149 | 26.61 | hsa-miR-590-3P_002677 | 24.79 | hsa-miR-589_001543 | 22.84 |
| hsa-miR-518f_002388 | 25.84 | hsa-miR-590-5p_001984 | 19.71 | hsa-miR-590-3P_002677 | 25.54 |
| hsa-miR-520c-3p_002400 | 24.65 | hsa-miR-597_001551 | 23.08 | hsa-miR-590-5p_001984 | 19.68 |
| hsa-miR-520e_001119 | 18.17 | hsa-miR-598_001988 | 26.51 | hsa-miR-597_001551 | 22.66 |
| hsa-miR-532_001518 | 16.53 | hsa-miR-601_001558 | 17.02 | hsa-miR-598_001988 | 25.74 |
| hsa-miR-532-3p_002355 | 18.07 | hsa-miR-605_001568 | 23.75 | hsa-miR-601_001558 | 19.77 |
| hsa-miR-539_001286 | 21.39 | hsa-miR-615-5p_002353 | 22.82 | hsa-miR-605_001568 | 22.51 |
| hsa-miR-542-3p_001284 | 21.47 | hsa-miR-616_001589 | 24.63 | hsa-miR-615-5p_002353 | 22.44 |
| hsa-miR-542-5p_002240 | 23.45 | hsa-miR-616_002414 | 24.82 | hsa-miR-616_001589 | 23.72 |
| hsa-miR-543_002376 | 21.42 | hsa-miR-624_001557 | 25.31 | hsa-miR-616_002414 | 23.83 |
| hsa-miR-545_002267 | 26.81 | hsa-miR-625#_002432 | 21.82 | hsa-miR-618_001593 | 21.41 |
| hsa-miR-548a_001538 | 26.90 | hsa-miR-625_002431 | 26.07 | hsa-miR-624_001557 | 25.84 |
| hsa-miR-548b-5p_002408 | 22.98 | hsa-miR-628-3p_002434 | 26.26 | hsa-miR-625#_002432 | 21.69 |
| hsa-miR-548c-5p_002429 | 23.26 | hsa-miR-628-5p_002433 | 25.40 | hsa-miR-625_002431 | 26.43 |
| hsa-miR-548d_001605 | 25.72 | hsa-miR-629_001562 | 22.83 | hsa-miR-628-3p_002434 | 26.07 |
| hsa-miR-548d-5p_002237 | 23.36 | hsa-miR-629_002436 | 26.75 | hsa-miR-628-5p_002433 | 25.74 |
| hsa-miR-550_002410 | 26.73 | hsa-miR-638_001582 | 20.81 | hsa-miR-629_001562 | 21.84 |
| hsa-miR-551b#_002346 | 25.01 | hsa-miR-642_001592 | 22.85 | hsa-miR-629_002436 | 26.78 |
| hsa-miR-572_001614 | 22.11 | hsa-miR-645_001597 | 21.01 | hsa-miR-635_001578 | 26.09 |
| hsa-miR-574-3p_002349 | 13.54 | hsa-miR-652_002352 | 22.64 | hsa-miR-638_001582 | 20.80 |
| hsa-miR-576-3p_002351 | 22.15 | hsa-miR-654_001611 | 18.43 | hsa-miR-641_001585 | 26.43 |
| hsa-miR-576-5p_002350 | 25.60 | hsa-miR-654-3p_002239 | 24.76 | hsa-miR-642_001592 | 22.55 |
| hsa-miR-579_002398 | 23.83 | hsa-miR-655_001612 | 24.65 | hsa-miR-645_001597 | 22.08 |
| hsa-miR-589_002409 | 26.59 | hsa-miR-660_001515 | 17.94 | hsa-miR-652_002352 | 23.24 |
| hsa-miR-590-3P_002677 | 24.71 | hsa-miR-661_001606 | 20.57 | hsa-miR-654_001611 | 18.14 |
| hsa-miR-590-5p_001984 | 19.27 | hsa-miR-663B_002857 | 16.87 | hsa-miR-654-3p_002239 | 24.11 |
| hsa-miR-591_001545 | 26.22 | hsa-miR-664_002897 | 20.22 | hsa-miR-655_001612 | 23.59 |
| hsa-miR-597_001551 | 22.45 | hsa-miR-671-3p_002322 | 22.41 | hsa-miR-656_001510 | 23.64 |
| hsa-miR-598_001988 | 23.85 | hsa-miR-708_002341 | 20.75 | hsa-miR-660_001515 | 17.76 |
| hsa-miR-601_001558 | 19.77 | hsa-miR-720_002895 | 10.79 | hsa-miR-661_001606 | 18.78 |
| hsa-miR-605_001568 | 22.62 | hsa-miR-744#_002325 | 25.22 | hsa-miR-663B_002857 | 15.58 |
| hsa-miR-616_001589 | 24.18 | hsa-miR-744_002324 | 18.82 | hsa-miR-664_002897 | 20.66 |
| hsa-miR-616_002414 | 23.78 | hsa-miR-758_001990 | 23.14 | hsa-miR-671-3p_002322 | 22.35 |
| hsa-miR-622_001553 | 23.95 | hsa-miR-766_001986 | 21.82 | hsa-miR-708_002341 | 20.84 |
| hsa-miR-624_001557 | 24.80 | hsa-miR-769-5p_001998 | 22.16 | hsa-miR-720_002895 | 11.18 |
| hsa-miR-625#_002432 | 21.83 | hsa-miR-885-5p_002296 | 26.37 | hsa-miR-744#_002325 | 24.30 |
| hsa-miR-625_002431 | 25.97 | hsa-miR-886-3p_002194 | 17.71 | hsa-miR-744_002324 | 19.69 |
| hsa-miR-628-3p_002434 | 25.91 | hsa-miR-886-5p_002193 | 18.77 | hsa-miR-758_001990 | 23.02 |
| hsa-miR-628-5p_002433 | 24.49 | hsa-miR-889_002202 | 22.83 | hsa-miR-766_001986 | 19.77 |
| hsa-miR-629_001562 | 22.44 | hsa-miR-92a_000431 | 13.23 | hsa-miR-769-5p_001998 | 22.36 |
| hsa-miR-629_002436 | 25.94 | hsa-miR-93#_002139 | 22.60 | hsa-miR-885-5p_002296 | 25.45 |
| hsa-miR-635_001578 | 26.31 | hsa-miR-942_002187 | 20.73 | hsa-miR-886-3p_002194 | 17.82 |
| hsa-miR-636_002088 | 19.75 | hsa-miR-95_000433 | 19.17 | hsa-miR-886-5p_002193 | 19.09 |
| hsa-miR-638_001582 | 20.74 | hsa-miR-99a#_002141 | 23.12 | hsa-miR-888_002212 | 23.75 |
| hsa-miR-642_001592 | 22.22 | hsa-miR-99a_000435 | 11.82 | hsa-miR-889_002202 | 22.31 |
| hsa-miR-645_001597 | 22.00 | hsa-miR-99b#_002196 | 22.44 | hsa-miR-9_000583 | 22.26 |
| hsa-miR-652_002352 | 21.67 | hsa-miR-99b_000436 | 12.88 | hsa-miR-92a_000431 | 12.92 |
| hsa-miR-654_001611 | 18.74 |  |  | hsa-miR-93#_002139 | 22.07 |
| hsa-miR-654-3p_002239 | 24.72 |  |  | hsa-miR-942_002187 | 20.72 |
| hsa-miR-655_001612 | 23.38 |  |  | hsa-miR-943_002188 | 24.27 |
| hsa-miR-656_001510 | 24.15 |  |  | hsa-miR-95_000433 | 19.58 |
| hsa-miR-660_001515 | 16.84 |  |  | hsa-miR-99a#_002141 | 22.76 |
| hsa-miR-663B_002857 | 17.28 |  |  | hsa-miR-99a_000435 | 12.62 |
| hsa-miR-664_002897 | 18.24 |  |  | hsa-miR-99b#_002196 | 22.66 |
| hsa-miR-671-3p_002322 | 22.43 |  |  | hsa-miR-99b_000436 | 13.79 |
| hsa-miR-708_002341 | 18.05 |  |  |  |  |
| hsa-miR-720_002895 | 11.81 |  |  |  |  |
| hsa-miR-744#_002325 | 23.90 |  |  |  |  |
| hsa-miR-744_002324 | 18.19 |  |  |  |  |
| hsa-miR-758_001990 | 23.24 |  |  |  |  |
| hsa-miR-769-5p_001998 | 21.61 |  |  |  |  |
| hsa-miR-876-3p_002225 | 26.81 |  |  |  |  |
| hsa-miR-886-3p_002194 | 17.71 |  |  |  |  |
| hsa-miR-886-5p_002193 | 18.39 |  |  |  |  |
| hsa-miR-888_002212 | 25.36 |  |  |  |  |
| hsa-miR-889_002202 | 23.20 |  |  |  |  |
| hsa-miR-92a_000431 | 12.78 |  |  |  |  |
| hsa-miR-93#_002139 | 21.82 |  |  |  |  |
| hsa-miR-942_002187 | 21.25 |  |  |  |  |
| hsa-miR-95_000433 | 20.45 |  |  |  |  |
| hsa-miR-99a#_002141 | 22.77 |  |  |  |  |
| hsa-miR-99a_000435 | 11.00 |  |  |  |  |
| hsa-miR-99b#_002196 | 21.93 |  |  |  |  |
| hsa-miR-99b_000436 | 12.31 |  |  |  |  |

Table S2. Shared miRNAs in ASC-EVs (normalized)

| miRNA | C_RT_ | SD |
| --- | --- | --- |
| hsa-miR-1274B_002884 | 7.62 | 1.01 |
| hsa-miR-125b_000449 | 9.14 | 0.37 |
| hsa-miR-24_000402 | 9.04 | 0.17 |
| hsa-miR-21_000397 | 10.50 | 0.38 |
| hsa-miR-222_002276 | 10.77 | 0.22 |
| hsa-miR-221_000524 | 10.60 | 0.53 |
| hsa-miR-1274A_002883 | 9.57 | 1.09 |
| hsa-miR-100_000437 | 11.30 | 0.46 |
| hsa-miR-99a_000435 | 11.31 | 0.45 |
| hsa-miR-193b_002367 | 11.05 | 0.05 |
| hsa-miR-30c_000419 | 11.53 | 0.16 |
| hsa-miR-31_002279 | 12.75 | 1.01 |
| hsa-miR-30b_000602 | 11.92 | 0.14 |
| hsa-miR-720_002895 | 10.75 | 0.93 |
| hsa-miR-145_002278 | 12.45 | 0.48 |
| hsa-miR-99b_000436 | 12.49 | 0.45 |
| hsa-miR-191_002299 | 12.48 | 0.01 |
| hsa-miR-20a_000580 | 12.62 | 0.30 |
| hsa-miR-92a_000431 | 12.47 | 0.33 |
| hsa-miR-29a_002112 | 13.17 | 0.33 |
| hsa-miR-19b_000396 | 12.88 | 0.26 |
| hsa-miR-27a_000408 | 13.18 | 0.23 |
| hsa-miR-214_002306 | 13.26 | 0.19 |
| hsa-miR-199a-3p_002304 | 13.30 | 0.33 |
| hsa-miR-574-3p_002349 | 13.37 | 0.18 |
| hsa-miR-30a-5p_000417 | 13.76 | 0.13 |
| hsa-miR-26a_000405 | 13.97 | 0.64 |
| hsa-miR-34a_000426 | 13.68 | 0.32 |
| hsa-miR-130a_000454 | 13.81 | 0.11 |
| hsa-miR-132_000457 | 14.20 | 0.46 |
| hsa-miR-29c_000587 | 14.34 | 0.46 |
| hsa-miR-218_000521 | 13.53 | 0.47 |
| hsa-miR-152_000475 | 14.08 | 0.18 |
| hsa-miR-328_000543 | 13.96 | 0.18 |
| hsa-miR-16_000391 | 14.32 | 0.16 |
| hsa-miR-320_002277 | 14.32 | 0.15 |
| hsa-miR-17_002308 | 14.32 | 0.39 |
| hsa-miR-106a_002169 | 14.46 | 0.57 |
| hsa-let-7a_000377 | 15.56 | 1.62 |
| hsa-miR-484_001821 | 14.42 | 0.06 |
| hsa-miR-127_000452 | 13.89 | 0.54 |
| hsa-miR-224_002099 | 14.03 | 0.34 |
| hsa-miR-31#_002113 | 14.72 | 0.30 |
| hsa-miR-331_000545 | 14.98 | 0.55 |
| hsa-miR-106b_000442 | 14.73 | 0.17 |
| hsa-miR-197_000497 | 14.41 | 0.33 |
| hsa-miR-382_000572 | 13.90 | 0.81 |
| hsa-miR-10a_000387 | 15.17 | 0.75 |
| hsa-miR-138_002284 | 15.54 | 0.63 |
| hsa-miR-27b_000409 | 14.64 | 0.36 |
| hsa-miR-181a_000480 | 16.39 | 1.42 |
| hsa-miR-365_001020 | 15.62 | 0.35 |
| hsa-miR-28_000411 | 16.02 | 0.47 |
| hsa-miR-409-3p_002332 | 14.98 | 0.62 |
| hsa-miR-22_000398 | 15.81 | 0.10 |
| hsa-miR-335_000546 | 16.56 | 0.73 |
| hsa-miR-26b_000407 | 15.97 | 0.45 |
| hsa-miR-193a-5p_002281 | 15.79 | 0.07 |
| hsa-miR-25_000403 | 15.50 | 0.46 |
| hsa-miR-210_000512 | 15.80 | 0.09 |
| hsa-miR-29b_000413 | 16.62 | 0.70 |
| hsa-let-7c_000379 | 15.61 | 0.53 |
| hsa-miR-143_002249 | 16.32 | 0.63 |
| hsa-miR-15b_000390 | 16.25 | 0.34 |
| hsa-miR-195_000494 | 16.16 | 0.24 |
| hsa-miR-296_000527 | 15.75 | 0.36 |
| hsa-miR-23a_000399 | 16.42 | 0.18 |
| hsa-miR-30d_000420 | 16.28 | 0.12 |
| hsa-miR-376a_000565 | 15.49 | 0.75 |
| hsa-miR-376c_002122 | 15.96 | 0.35 |
| hsa-miR-324-5p_000539 | 16.92 | 0.48 |
| hsa-miR-30e-3p_000422 | 16.31 | 0.21 |
| hsa-miR-28-3p_002446 | 16.41 | 0.04 |
| hsa-miR-532_001518 | 16.78 | 0.23 |
| hsa-miR-1260_002896 | 15.72 | 0.76 |
| hsa-miR-30a-3p_000416 | 16.52 | 0.11 |
| hsa-miR-149_002255 | 16.31 | 0.29 |
| hsa-miR-148a_000470 | 16.70 | 0.16 |
| hsa-miR-361_000554 | 16.63 | 0.14 |
| hsa-miR-374_000563 | 17.41 | 0.70 |
| hsa-miR-660_001515 | 17.01 | 0.19 |
| hsa-miR-103_000439 | 18.12 | 1.22 |
| hsa-miR-10b_002218 | 17.71 | 1.07 |
| hsa-miR-34a#_002316 | 16.74 | 0.18 |
| hsa-miR-130b_000456 | 16.83 | 0.18 |
| hsa-miR-1291_002838 | 17.09 | 0.06 |
| hsa-miR-411_001610 | 17.77 | 0.69 |
| hsa-miR-663B_002857 | 16.07 | 1.25 |
| hsa-miR-29a#_002447 | 17.25 | 0.28 |
| hsa-miR-424_000604 | 18.01 | 0.79 |
| hsa-miR-10b#_002315 | 17.38 | 0.25 |
| hsa-miR-34c_000428 | 17.60 | 0.19 |
| hsa-miR-886-3p_002194 | 17.24 | 0.41 |
| hsa-miR-125a-5p_002198 | 17.89 | 0.24 |
| hsa-miR-186_002285 | 18.03 | 0.26 |
| hsa-miR-342-3p_002260 | 17.56 | 0.25 |
| hsa-miR-204_000508 | 18.06 | 1.24 |
| hsa-miR-146b_001097 | 18.18 | 0.38 |
| hsa-miR-212_000515 | 18.32 | 0.44 |
| hsa-let-7g_002282 | 18.06 | 0.53 |
| hsa-miR-151-3p_002254 | 18.33 | 0.66 |
| hsa-miR-483-5p_002338 | 16.89 | 1.00 |
| hsa-miR-199a_000498 | 18.28 | 0.36 |
| hsa-miR-708_002341 | 19.37 | 1.15 |
| hsa-miR-532-3p_002355 | 18.56 | 0.43 |
| hsa-miR-34b_002102 | 17.87 | 0.34 |
| hsa-miR-744_002324 | 18.39 | 0.44 |
| hsa-miR-664_002897 | 19.20 | 0.85 |
| hsa-miR-886-5p_002193 | 18.25 | 0.18 |
| hsa-miR-19a_000395 | 18.24 | 0.31 |
| hsa-miR-199b_000500 | 18.06 | 0.42 |
| hsa-miR-22#_002301 | 18.29 | 0.23 |
| hsa-miR-423-5p_002340 | 18.26 | 0.29 |
| hsa-let-7d_002283 | 19.53 | 1.22 |
| hsa-miR-410_001274 | 18.43 | 0.28 |
| hsa-miR-301_000528 | 18.59 | 0.08 |
| hsa-miR-654_001611 | 17.93 | 0.72 |
| hsa-miR-424#_002309 | 18.73 | 0.12 |
| hsa-miR-494_002365 | 18.73 | 0.21 |
| hsa-miR-455-3p_002244 | 20.03 | 0.96 |
| hsa-miR-590-5p_001984 | 19.05 | 0.20 |
| hsa-miR-192_000491 | 19.70 | 0.39 |
| hsa-miR-320B_002844 | 19.20 | 0.26 |
| hsa-miR-324-3p_002161 | 19.67 | 0.18 |
| hsa-miR-222#_002097 | 19.10 | 0.57 |
| hsa-miR-15a_000389 | 20.54 | 0.88 |
| hsa-miR-146a_000468 | 21.37 | 1.82 |
| hsa-miR-503_001048 | 19.30 | 0.39 |
| hsa-miR-452_002329 | 19.72 | 0.26 |
| hsa-miR-601_001558 | 18.35 | 1.82 |
| hsa-miR-330_000544 | 20.25 | 0.43 |
| hsa-miR-24-2#_002441 | 20.02 | 0.23 |
| hsa-miR-193b#_002366 | 19.76 | 0.17 |
| hsa-miR-345_002186 | 20.02 | 0.20 |
| hsa-miR-128a_002216 | 19.99 | 0.18 |
| hsa-miR-214#_002293 | 20.52 | 0.28 |
| hsa-miR-148b_000471 | 20.75 | 0.31 |
| hsa-miR-455_001280 | 20.95 | 0.52 |
| hsa-miR-95_000433 | 19.23 | 1.07 |
| hsa-miR-155_002623 | 21.11 | 0.55 |
| hsa-miR-185_002271 | 20.34 | 0.23 |
| hsa-miR-125b-1#_002378 | 20.42 | 0.36 |
| hsa-miR-184_000485 | 21.07 | 0.78 |
| hsa-miR-638_001582 | 20.28 | 0.40 |
| hsa-miR-500_002428 | 21.54 | 0.68 |
| hsa-miR-190_000489 | 21.61 | 1.12 |
| hsa-miR-181c_000482 | 22.16 | 1.10 |
| hsa-miR-140-3p_002234 | 21.43 | 0.43 |
| hsa-miR-18a_002422 | 20.73 | 0.60 |
| hsa-miR-151-5P_002642 | 21.80 | 0.57 |
| hsa-miR-154_000477 | 21.20 | 0.22 |
| hsa-miR-339-3p_002184 | 21.09 | 0.12 |
| hsa-miR-942_002187 | 20.39 | 0.74 |
| hsa-miR-194_000493 | 22.78 | 1.24 |
| hsa-miR-505_002089 | 21.48 | 0.51 |
| hsa-miR-539_001286 | 21.16 | 0.28 |
| hsa-miR-543_002376 | 20.34 | 0.94 |
| hsa-miR-126_002228 | 23.14 | 1.51 |
| hsa-miR-497_001043 | 21.10 | 0.51 |
| hsa-miR-542-3p_001284 | 21.83 | 0.44 |
| hsa-miR-454_002323 | 21.81 | 1.08 |
| hsa-miR-1303_002792 | 21.52 | 0.70 |
| hsa-miR-769-5p_001998 | 21.54 | 0.09 |
| hsa-miR-101_002253 | 22.06 | 0.45 |
| hsa-miR-20b_001014 | 21.65 | 1.18 |
| hsa-miR-652_002352 | 22.01 | 0.40 |
| hsa-miR-502-3p_002083 | 21.55 | 0.35 |
| hsa-miR-323-3p_002227 | 20.75 | 0.87 |
| hsa-miR-34b_000427 | 21.67 | 0.09 |
| hsa-miR-433_001028 | 21.35 | 0.46 |
| hsa-miR-150_000473 | 22.78 | 1.20 |
| hsa-miR-487b_001285 | 21.12 | 0.56 |
| hsa-miR-485-3p_001277 | 20.96 | 0.79 |
| hsa-miR-203_000507 | 22.18 | 0.45 |
| hsa-miR-1290_002863 | 21.08 | 0.63 |
| hsa-miR-502_001109 | 22.43 | 0.59 |
| hsa-miR-337-5p_002156 | 21.17 | 0.57 |
| hsa-miR-362_001273 | 22.82 | 0.94 |
| hsa-miR-93#_002139 | 21.66 | 0.33 |
| hsa-miR-625#_002432 | 21.27 | 0.49 |
| hsa-miR-139-5p_002289 | 21.91 | 0.40 |
| hsa-miR-99b#_002196 | 21.84 | 0.11 |
| hsa-miR-181a-2#_002317 | 22.99 | 0.96 |
| hsa-miR-645_001597 | 21.19 | 0.86 |
| hsa-miR-196b_002215 | 22.74 | 1.04 |
| hsa-miR-572_001614 | 21.75 | 0.36 |
| hsa-miR-432_001026 | 20.99 | 1.02 |
| hsa-miR-576-3p_002351 | 22.17 | 0.16 |
| hsa-miR-642_001592 | 22.03 | 0.24 |
| hsa-miR-136#_002100 | 21.62 | 0.55 |
| hsa-miR-1271_002779 | 21.28 | 1.17 |
| hsa-miR-671-3p_002322 | 21.89 | 0.47 |
| hsa-miR-629_001562 | 21.86 | 0.73 |
| hsa-miR-597_001551 | 22.22 | 0.31 |
| hsa-miR-409-5p_002331 | 22.86 | 0.50 |
| hsa-miR-1285_002822 | 22.43 | 1.55 |
| hsa-miR-381_000571 | 21.82 | 0.69 |
| hsa-miR-605_001568 | 22.46 | 0.67 |
| hsa-miR-27a#_002445 | 22.41 | 0.35 |
| hsa-miR-493_002364 | 22.49 | 0.35 |
| hsa-miR-223_002295 | 23.37 | 0.53 |
| hsa-miR-99a#_002141 | 22.38 | 0.40 |
| hsa-miR-1255B_002801 | 22.66 | 1.16 |
| hsa-miR-301b_002392 | 23.07 | 0.16 |
| hsa-miR-548b-5p_002408 | 22.88 | 0.65 |
| hsa-miR-1226#_002758 | 22.10 | 0.96 |
| hsa-miR-299-5p_000600 | 22.93 | 0.29 |
| hsa-miR-1180_002847 | 22.15 | 0.88 |
| hsa-miR-889_002202 | 22.27 | 0.85 |
| hsa-miR-145#_002149 | 23.18 | 0.38 |
| hsa-miR-758_001990 | 22.63 | 0.54 |
| hsa-miR-548c-5p_002429 | 23.28 | 0.54 |
| hsa-miR-329_001101 | 22.63 | 0.65 |
| hsa-miR-548d-5p_002237 | 23.56 | 0.59 |
| hsa-miR-198_002273 | 23.58 | 1.25 |
| hsa-miR-369-3p_000557 | 22.59 | 0.82 |
| hsa-miR-655_001612 | 23.37 | 0.57 |
| hsa-miR-362-3p_002117 | 23.07 | 0.31 |
| hsa-miR-542-5p_002240 | 23.78 | 0.30 |
| hsa-miR-126#_000451 | 24.53 | 0.83 |
| hsa-miR-27b#_002174 | 23.08 | 0.81 |
| hsa-miR-378_002243 | 22.89 | 0.85 |
| hsa-miR-154#_000478 | 23.61 | 0.06 |
| hsa-miR-505#_002087 | 23.82 | 0.14 |
| hsa-miR-221#_002096 | 24.50 | 0.67 |
| hsa-miR-340_002258 | 23.89 | 0.38 |
| hsa-miR-616_002414 | 23.64 | 0.55 |
| hsa-miR-579_002398 | 23.17 | 0.62 |
| hsa-miR-598_001988 | 24.86 | 0.97 |
| hsa-miR-744#_002325 | 23.97 | 0.50 |
| hsa-miR-296-3p_002101 | 23.59 | 0.34 |
| hsa-miR-425#_002302 | 23.88 | 0.21 |
| hsa-miR-450a_002303 | 24.30 | 0.45 |
| hsa-miR-376b_001102 | 23.36 | 0.65 |
| hsa-miR-1300_002902 | 24.37 | 0.29 |
| hsa-miR-146b-3p_002361 | 24.34 | 0.24 |
| hsa-miR-616_001589 | 23.67 | 0.66 |
| hsa-miR-372_000560 | 24.77 | 1.08 |
| hsa-miR-337-3p_002157 | 23.58 | 0.69 |
| hsa-miR-15a#_002419 | 24.92 | 1.12 |
| hsa-miR-340#_002259 | 25.43 | 0.86 |
| hsa-miR-487a_001279 | 24.11 | 0.37 |
| hsa-miR-628-5p_002433 | 24.71 | 0.23 |
| hsa-miR-373_000561 | 23.92 | 0.64 |
| hsa-miR-148b#_002160 | 24.56 | 0.49 |
| hsa-miR-23a#_002439 | 24.78 | 0.60 |
| hsa-miR-590-3P_002677 | 24.51 | 0.38 |
| hsa-miR-654-3p_002239 | 24.03 | 0.70 |
| hsa-miR-10a#_002288 | 24.58 | 0.58 |
| hsa-miR-624_001557 | 24.81 | 0.23 |
| hsa-miR-378_000567 | 25.02 | 0.91 |
| hsa-miR-380-5p_000570 | 23.55 | 1.10 |
| hsa-miR-30d#_002305 | 24.85 | 0.41 |
| hsa-miR-125a-3p_002199 | 25.42 | 0.46 |
| hsa-miR-377#_002128 | 25.02 | 0.16 |
| hsa-miR-19b-1#_002425 | 24.89 | 0.50 |
| hsa-miR-16-1#_002420 | 25.30 | 0.32 |
| hsa-miR-20a#_002437 | 25.80 | 0.46 |
| hsa-miR-33a#_002136 | 25.82 | 0.31 |
| hsa-miR-375_000564 | 24.93 | 0.63 |
| hsa-miR-206_000510 | 25.85 | 0.22 |
| hsa-miR-32_002109 | 25.76 | 0.31 |
| hsa-miR-1270_002807 | 25.53 | 0.44 |
| hsa-miR-628-3p_002434 | 25.57 | 0.32 |
| hsa-miR-629_002436 | 25.98 | 0.04 |
| hsa-miR-625_002431 | 25.65 | 0.31 |
| hsa-miR-18a#_002423 | 25.78 | 0.72 |

Table S3. Top 63 ASC-EVs embedded-miRNAs falling in the first quartile of expression

| hsa-let-7a | hsa-miR-30b |
| --- | --- |
| hsa-let-7c | hsa-miR-30c |
| hsa-miR-100 | hsa-miR-30d |
| hsa-miR-106a | hsa-miR-31 |
| hsa-miR-106b | hsa-miR-320 |
| hsa-miR-10a | hsa-miR-328 |
| hsa-miR-125b | hsa-miR-331 |
| hsa-miR-1260 | hsa-miR-34a |
| hsa-miR-127 | hsa-miR-365 |
| hsa-miR-130a | hsa-miR-376a |
| hsa-miR-132 | hsa-miR-376c |
| hsa-miR-138 | hsa-miR-382 |
| hsa-miR-145 | hsa-miR-409-3p |
| hsa-miR-152 | hsa-miR-484 |
| hsa-miR-15b | hsa-miR-574-3p |
| hsa-miR-16 | hsa-miR-663b |
| hsa-miR-17 | hsa-miR-92a |
| hsa-miR-191 | hsa-miR-99a |
| hsa-miR-193a-5p | hsa-miR-99b |
| hsa-miR-193b |  |
| hsa-miR-195 |  |
| hsa-miR-197 |  |
| hsa-miR-199a-3p |  |
| hsa-miR-19b |  |
| hsa-miR-20a |  |
| hsa-miR-21 |  |
| hsa-miR-210 |  |
| hsa-miR-214 |  |
| hsa-miR-218 |  |
| hsa-miR-22 |  |
| hsa-miR-221 |  |
| hsa-miR-222 |  |
| hsa-miR-224 |  |
| hsa-miR-24 |  |
| hsa-miR-25 |  |
| hsa-miR-26a |  |
| hsa-miR-26b |  |
| hsa-miR-27a |  |
| hsa-miR-27b |  |
| hsa-miR-28 |  |
| hsa-miR-296 |  |
| hsa-miR-29a |  |
| hsa-miR-29c |  |
| hsa-miR-30a-5p |  |

Table S4. ASC-EVs miRNAs in the first quartile of expression and OA-related. [52]

| miRNA | Chondrogenesis | Cartilage degradation | Osteoclastogenesis | Osteoblastogenesis | Role |
| --- | --- | --- | --- | --- | --- |
| miR-15b |  |  |  | **+** | Induces osteoblast differentiation |
| miR-20a |  |  |  | **-** | Reduces osteoblast differentiation |
| miR-21 |  |  | **+** |  | Attenuates the process of chondrogenesis, increases in aging cartilage |
| miR-22 |  | **+** |  |  | Upregulated in OA cartilage |
| miR-26a |  |  |  | **+** | Modulates late osteoblast differentiation, inhibitor of osteoblast differentiation, downregulated in OA cartilage |
| miR-26b | **+** |  |  |  | Upregulated during chondrogenesis |
| miR-27b |  | **-** |  |  | Reduces the degradation of cartilage |
| miR-28 | **+** |  |  |  | Upregulated during chondrogenesis |
| miR-29a |  |  |  | **+** | Enhances osteoblastogenesis, downregulated during OA |
| miR-100 |  |  |  | **-** | Negative role in osteogenic differentiation by targeting BMPR2 directly |
| miR-125b |  | **-** |  |  | Inhibitor of osteoblastic differentiation, inhibits normal chondrocyte ECM degradation |
| miR-145 |  | **+** |  |  | Negatively regulates chondrogenesis |
| miR-152 | **+** |  |  |  | Upregulated during chondrogenesis |
| miR-210 |  |  |  | **+** | Positive regulator of osteoblast, chondrogenesis differentiation, downregulated in OA cartilage |
| miR-214 |  |  |  | **-** | Suppresses the osteogenic differentiation, inhibit osteoblast activity |
| miR-218 |  |  |  | **+** | Enhances osteoblast differentiation |
| miR-221 |  |  |  | **-** | Inhibits osteoblast differentiation |

Table S5. Targets expressed in IL1β treated FLSs for 11 miRNAs present only in ASC-EVs

| *ACTR1A* | *OCIAD1* |
| --- | --- |
| *AK2* | *PAICS* |
| *APP* | *PAPOLA* |
| *ARF1* | *PFN2* |
| *ASCC2* | *PITX1* |
| *BTG1* | *PLS3* |
| *CALU* | *POM121* |
| *CAPNS1* | *PPT1* |
| *CCND1* | *PRDX3* |
| *CCND2* | *PRNP* |
| *CCND3* | *PSAP* |
| *CDK6* | *PSME1* |
| *CREB1* | *PSMF1* |
| *CRKL* | *PTP4A2* |
| *CS* | *PTPN4* |
| *DCBLD2* | *PUDP* |
| *DEK* | *PURB* |
| *DNAJB6* | *RAB5B* |
| *EIF3L* | *RANGAP1* |
| *EXT1* | *RCC2* |
| *EZR* | *RHOC* |
| *FAM213A* | *RPL36* |
| *FKBP14* | *SERF1B* |
| *FKBP1A* | *SH3PXD2A* |
| *GAPDH* | *SLC38A2* |
| *GMFB* | *SLC7A1* |
| *GNAI2* | *SNRPB* |
| *GNS* | *SOD2* |
| *GRSF1* | *STX16* |
| *HNRNPM* | *SYF2* |
| *IL6* | *SZRD1* |
| *ITM2B* | *TMEM59* |
| *KCTD5* | *TOMM20* |
| *KDELR1* | *TPI1* |
| *KLF6* | *TRAF3IP2* |
| *KPNA4* | *TXNIP* |
| *KPNB1* | *UBA52* |
| *LDLR* | *VAMP3* |
| *LPP* | *YIPF6* |
| *MCM4* | *ZMYND11* |
| *MCRS1* |  |
| *NFKB1* |  |
| *NOP2* |  |
| *NPM1* |  |
